# Covalent Benzenesulfonic Functionalization of a Graphene Nanopore for Enhanced and Selective Proton Transport

**DOI:** 10.1021/acs.jpcc.3c07406

**Published:** 2024-02-21

**Authors:** Dario Calvani, Bas Kreupeling, G. J. Agur Sevink, Huub J. M. de Groot, Grégory F. Schneider, Francesco Buda

**Affiliations:** † Leiden Institute of Chemistry, Leiden University, P.O. Box 9502, 2300 RA Leiden, The Netherlands

## Abstract

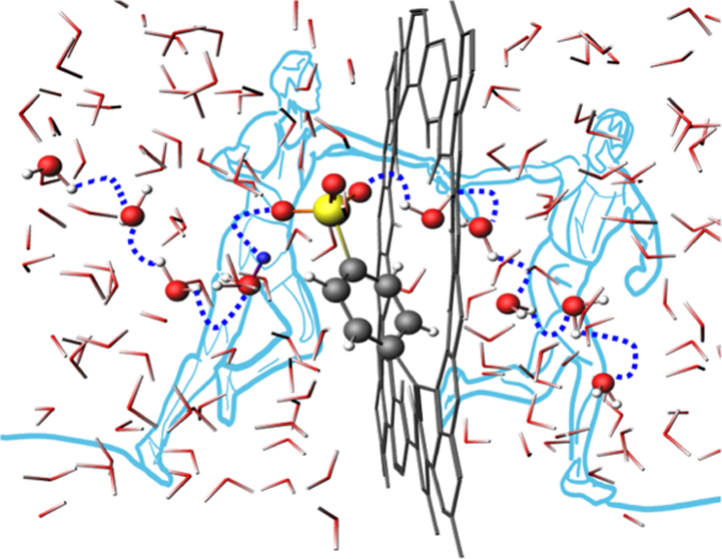

A fundamental understanding of proton transport through graphene nanopores, defects, and vacancies is essential for advancing two-dimensional proton exchange membranes (PEMs). This study employs ReaxFF molecular dynamics, metadynamics, and density functional theory to investigate the enhanced proton transport through a graphene nanopore. Covalently functionalizing the nanopore with a benzenesulfonic group yields consistent improvements in proton permeability, with a lower activation barrier (≈0.15 eV) and increased proton selectivity over sodium cations. The benzenesulfonic functionality acts as a dynamic proton shuttle, establishing a favorable hydrogen-bonding network and an efficient proton transport channel. The model reveals an optimal balance between proton permeability and selectivity, which is essential for effective proton exchange membranes. Notably, the benzenesulfonic-functionalized graphene nanopore system achieves a theoretically estimated proton diffusion coefficient comparable to or higher than the current state-of-the-art PEM, Nafion. Ergo, the benzenesulfonic functionalization of graphene nanopores, firmly holds promise for future graphene-based membrane development in energy conversion devices.

## 1 Introduction

Nowadays, the role of graphene and two-dimensional (2D) materials as ion-selective membranes is intensively investigated.^1,2^ The use of these atom-thick membranes finds its essence in several applications from energy conversion and storage technologies to desalination devices.^3−5^ The intrinsic properties of graphene as the atom-thickness, strength, and controllable surface chemistry enabled original routes for attractive functionalization in terms of selectivity, permeability, and enhanced ion transport.^2,6^ One aspect currently under significant debate due to its technological relevance is the transport of protons through graphene.^7^ A recent elegant experimental work by Bentley and co-workers draws an accurate and clear picture of the proton transport through high-quality (exfoliated or chemical vapor deposition, CVD) graphene, pointing out that protons mostly go through subnanometer defects with good proton permeability.^8^ On the contrary, proton transport is highly unfavorable through pristine graphene,^8^ somewhat in contrast with previous findings by Hu and co-workers.^9^ It has been shown computationally that a hydrogenated sp^3^ defect in pristine graphene can already lower the barrier for proton transport, thus slightly increasing the permeability.^10−12^ Other studies highlighted that the presence of defects as pores and their functionalization represents an attractive perspective to significantly improve the energetics of the proton transport process via an enhanced Grotthuss mechanism.^13−17^ Bukola and co-workers explored the selectivity for proton and other cations in Nafion–graphene–Nafion sandwich membranes.^18^ They observed that high proton selectivity relative to other cations occurs at certain defect sites, although the exact nature of these defects is still unclear.^18^ In general, an important requisite in the design of an efficient graphene-based proton exchange membrane (PEM) is to find an optimal balance between the proton permeability and selectivity. To achieve this goal, the implementation of sulfonic functionality^19−21^ orthogonally to the graphene basal plane (10.48550/arXiv.2308.16112) or at the edge of a nanopore appears as an attractive strategy. Experimentally, a tangible nonoxidative route for modification of the graphene surface and edges with a sulfonic-based group can be achieved by organo-radicals.^22−26^

**Figure 1:**
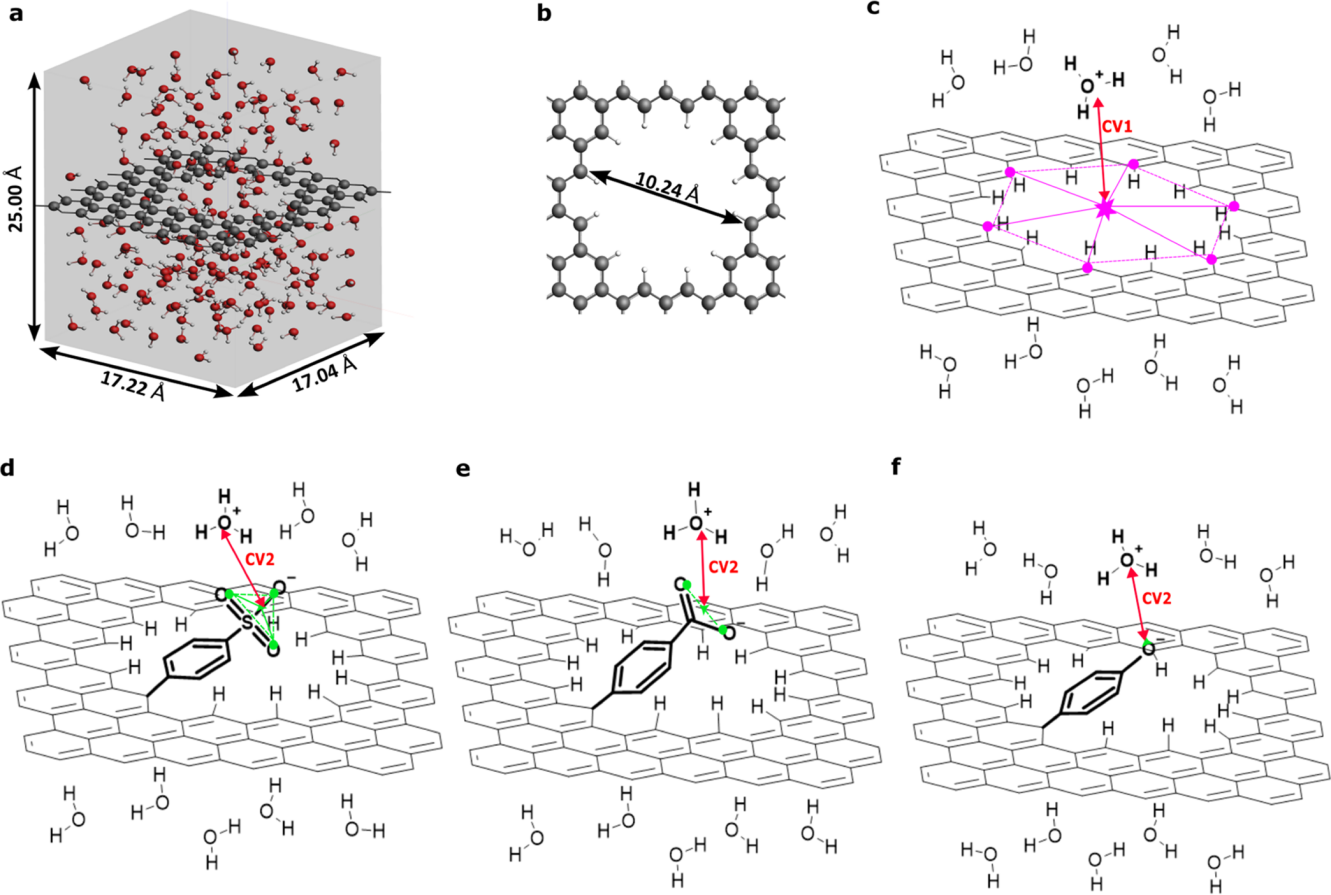
(a) Representative balls and sticks snapshot of the hydrogenated graphene nanopore solvated in water, with the simulation box represented in gray. (b) The hydrogenated graphene nanopore with a diameter of ≈10 Å. Carbon, oxygen, and hydrogen atoms are depicted in gray, red, and white balls, respectively. (c) Schematic representation of the hydrogenated graphene nanopore showing a few water molecules and one hydronium to represent the bulk water on both sides of the graphene. The collective variable CV1, indicated in red, represents the distance between the oxygen atom of the hydronium ion and the center of mass of the graphene nanopore along the *z*-axis. The center of mass is calculated using the position of the carbon atoms highlighted in purple. (d–f) Schematic representations of the same hydrogenated graphene nanopore with one hydrogen now being replaced by covalent functionalization with benzenesulfonic, benzoic, and phenol groups, respectively. The collective variable CV2, in red, is designed to describe the proton traveling from the hydronium in the water bulk to the center of mass of the oxygens (in green) of each anionic functional group (Ph-SO_3_^–^, Ph-COO^–^, Ph-O^–^), and *vice versa* from the oxygens (in green) of each functional group (Ph-SO_3_H, Ph-COOH, Ph-OH) to the water bulk.

In this work, it has been computationally investigated whether this functionalization can facilitate the Grotthuss-like proton hopping process through a graphene nanopore by leveraging its inherent acid/base properties^27,28^ and increasing the selectivity of a graphene nanopore by molecular steric hindrance.^13^ This model considers a simulation box with a hydrogenated graphene nanopore with a diameter of approximately 10 Å solvated in water, as shown in Figure 1a,b. The size and shape of the nanopore are consistent with a range of vacancies experimentally detected^29,30^ and that can also be produced via an electron beam technique with a specific intensity distribution^31^ or with an ion bombardment plasma procedure.^32−35^ Experimentally, the proton transfer through the membrane is driven either by an applied potential or a proton concentration gradient.^36^ In our simulations, no explicit driving force is included. This fact does not represent a limitation since the main goal of this study is to explore the fundamental mechanism at the base of proton transport.

First, proton transport through the hydrogenated graphene nanopore was studied (Figure 1c). Then, the same hydrogenated system was covalently functionalized at the inner edge of the graphene nanopore using different groups, each representing an increasing p*K*_a_ value: benzenesulfonic acid (Ph-SO_3_H), benzoic acid (Ph-COOH), and phenol (Ph-OH), as shown in Figure 1d,e,f. Proton transport is explored using ReaxFF force field molecular dynamics (ReaxFF-MD)^37−39^ and metadynamics^40^ simulations. This combination enables accelerating the crossing of (high) energy barriers concerning the unbiased MD simulations and estimates the free energy profile of this rare event.^41^ These simulations show the superiority of the benzenesulfonic-functionalized graphene nanopore over the other systems considered in both permeability and selectivity. The analysis of the MD trajectories elucidates the proton transport mechanism and the role of the benzenesulfonic group as a proton shuttle through the graphene nanopore. Moreover, the theoretically estimated proton diffusion coefficient across the graphene nanopore for the benzenesulfonic-functionalized system is found to be comparable to or larger than that of Nafion.^8,42,43^ Hereby, this research envisages the design of new high-performance graphene-based proton exchange membranes (PEMs) for potential applications in efficient electrochemical conversion (e.g., fuel cells)^7^ and energy storage devices (e.g., batteries),^3^ playing a crucial role toward the green energy transition.^44,45^

## 2 Computational Methods

### 2.1 ReaxFF-MD and Metadynamics Simulations

The ReaxFF-MD simulations are performed using the AMS2021 suite^46,47^ and the CHONSMgPNaTiClFKLi.ff force field^48,49^ suitable for describing these systems in a water environment.^50^ The CHONSMgPNaTiClFKLi.ff force field^48,49^ is further validated with the estimation of p*K*_a_ for benzenesulfonic acid in the water environment (see the Supporting Information, Section S1.1). Each starting system is built up from a 17.22 × 17.04 Å^2^ graphene layer placed in a cell of 17.22 × 17.04 × 25.00 Å^3^ dimensions (Figure 1a). A nanopore of ∼10 Å diameter is created and saturated with hydrogens (Figure 1b,c). In the functionalized systems, the functional group replaces one of the hydrogens at the graphene nanopore edge (Figure 1d,e,f). Each system is solvated with 170 water molecules to reach a total density of ∼1.0 g mL^–1^. Periodic boundary conditions (PBCs) are applied for each simulation. ReaxFF-MD equilibrations are performed for 0.5 ns each, with a time step of 0.25 fs and a damping constant of 1000 and 100 for pressure and temperature, respectively. First, an NPT equilibration run is performed at 300 K and 1 atm using a Martyna–Tobias–Klein (MTK) barostat and a Nosé–Hoover chain (NHC) thermostat. Second, the systems are further equilibrated in the NVT ensemble with the NHC thermostat at 300 K. The dimension of the simulation cell for the NPT equilibration is kept constant along the *x*- and *y*-axis (17.22 × 17.04 Å^2^ parallel to the graphene sheet) and changes only along the *z*-axis in the direction perpendicular to the sheet to 21.66 and 23.50 Å for the hydrogenated and functionalized systems, respectively. The total charges are equal to 0 and −1 for the hydrogenated and covalent-functionalized systems, respectively. After a proton is introduced into the bulk of the hydrogenated graphene nanopore system and into each functional group within the covalently functionalized graphene nanopore, the total charge reaches 1 and 0, respectively. Subsequently, an additional 0.5 ns NVT equilibration is conducted using the NHC thermostat at 300 K, employing a time step of 0.25 fs and a damping constant of 100 for temperature control. Finally, for each system, three independent NVT ReaxFF-MD plus metadynamics simulations, of 1 ns each, are performed employing the PLUMED plugin^51^ for the metadynamics, NHC thermostat, a temperature of 300 K, a time step of 0.25 fs, and a damping constant of 100 for the temperature. A well-tempered metadynamics method is employed with width = 0.5 Å, height = 0.1 kJ mol^–1^, bias factor = 10, and deposition frequency of Gaussian hills every 100 time steps per each ReaxFF-MD metadynamics simulation.^52^

To assess the convergence of a metadynamics simulation and to extrapolate each value of free energy reported for each system, the mean free energy value is estimated as a function of simulation time every 100 Gaussian kernels deposited as reported in Figures S4, S10, S12, S14, and S18, and the global minimum is set to zero in all profiles.

CV1 involved in the description of the proton transport through a hydrogenated graphene nanopore is defined as the distance *L*_1_ along the *z*-axis between the oxygen atom in the hydronium ion *r*_O_ (*z*) and the center of mass of the graphene nanopore *g*(*z*):^16^
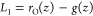
with
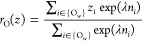
where *z*_*i*_ is the *z*-component of the distance from the oxygen *i* to the center of mass of the graphene nanopore, λ is a large constant, {O_W_} is the set of all oxygen atoms that belong to the water bulk, and *n*_*i*_ is the number of hydrogen atoms coordinated to oxygen *i*. The constant λ is set to 100 in the calculations. The function *L*_1_ approximates the distance between the hydronium ion and the center of mass of the graphene nanopore by identifying which oxygen has the most hydrogen atoms coordinated and returning the corresponding distance. The coordination number *n*_*i*_ can be calculated with
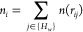
where {*H*_w_} is the set of all hydrogen atoms present in the bulk water. The switching function *n*(*r*_*ij*_) determines whether there is contact between two atoms and is written as
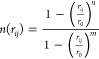
where *r*_*ij*_ is the distance between the oxygen atom *i* and hydrogen atom *j* of the bulk water. A cutoff distance *r*_0_ of 1.35 Å is used throughout all simulations, and the variables *n* and *m* are set to be 10 and 20, respectively. The coordination number *n*_*i*_ gets close to 3 when the oxygen atom *i* belongs to a hydronium ion and 2 in the case of water.

CV2 used for describing the proton traveling from the functional group into the bulk water for each functionalized system is defined as the distance *L*_2_ from the center of mass of the oxygens in each functional group (Ph-SO_3_^–^/Ph-SO_3_H, Ph-COO^–^/Ph-COOH, Ph-O^–^/Ph-OH) to the oxygen atom in the hydronium ion/water molecule coordinated to the proton in the functional group.^53^ This is identified as in CV1.
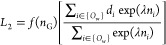
where *d*_*i*_ = |*r*_*i*_ – *r*_G_| is the distance between oxygen atom *i* and the center of mass of the oxygen atoms belonging to the functional group and *n*_G_ is the number of hydrogen atoms coordinated to the functional group. The switching function *f*(*n*_G_) included in CV2 is given by
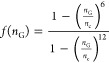
where the coordination cutoff *n*_c_ is a parameter, whose value depends on how many excess protons are present in the system; in this case, only one excess proton is present. The switching function *f*(*n*_G_) is included in CV2 since the distance calculated by the function in square brackets would not be meaningful if the functional group is protonated. For instance, when the functional groups are protonated (region I) in each ReaxFF-MD metadynamics simulation, the value of CV2 is near zero since *n*_G_ = 1 and *f*(*n*_G_) ≈ 0, and consequently, CV2 is close to zero. In region II and region III, the switching function *f*(*n*_G_) ≈ 1 results in CV2 depending on the value in square brackets and describing the distance from the center of mass of the oxygens in each functional group (Ph-SO_3_^–^/Ph-SO_3_H, Ph-COO^–^/Ph-COOH, Ph-O^–^/Ph-OH) to the oxygen atom in the hydronium ion/water molecule coordinated to the proton in the functional group.

CV3 is defined as the difference between the *z*-component of the center of mass of the oxygens of the benzenesulfonic functionality and the center of mass of the graphene nanopore.

Following the CV1 definition above, CV1* is defined as the distance *L*_1_^*^ along the *z*-axis between the sodium cation *r*_Na_(*z*) and the center of mass of the graphene nanopore *g*(*z*).
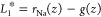


### 2.2 Density Functional Theory (DFT) Estimation of Proton Affinities (PAs)

The proton affinities (PAs) for the benzenesulfonic acid, benzoic, and phenol, connected to the different functional groups considered in this study (Ph-SO_3_H, Ph-COOH, and Ph-OH), were computed using DFT within the AMS2021 suite,^46,47^ with the hybrid B3LYP functional,^54−57^ including D3(BJ) dispersion correction^58,59^ and the TZP basis set, both in vacuum and in a COSMO-water-type solvation.^60^ The PAs are determined for the explicative reaction A^–^ + H^+^ → AH at a temperature of 0 K.^61^ See the Supporting Information, Section S2.4 and Table S1, for the PA estimations results.

## 3 Results and Discussion

### 3.1 Proton and Sodium Cation Transport through a Hydrogenated Graphene Nanopore in an Aqueous Environment

In earlier MD simulations involving solvated graphene-based systems, the graphene layer was maintained rigid.^14−16^ Here, a flexible graphene layer is considered to improve the reliability of the model (see the Supporting Information, Section S2.1, for comparison with the case of rigid graphene). A graphene layer with an ∼10 Å diameter hydrogenated nanopore fully solvated in water was built up, as depicted in Figure 1a,b. After the system was equilibrated (see Section 2 Computational Methods for the details), an excess proton solvated in water was introduced. Subsequently, three separate ReaxFF-MD metadynamics simulations, each lasting 1.0 ns, were conducted to estimate the averaged free energy along the collective variable CV1, as illustrated in Figure 1c (see Section 2 Computational Methods for the CV1 definition). This collective variable effectively describes the motion of the excess proton through the hydrogenated graphene nanopore. The free energy profile for proton transport gives an activation barrier of 22.3 ± 2.5 kJ mol^–1^ corresponding to the proton moving through the center of the nanopore (CV1 ≈ 0 Å) (see the Supporting Information, Section S2.1, Figures S3–S5). The activation barrier for the rigid case is 11.6 ± 1.1 kJ mol^–1^ and lower than that for the flexible case likely due to entropic effects of the graphene sheet fluctuations. Moreover, it has been found that ∼12% of the water molecules access the nanopore region for both the flexible and rigid graphene cases (see the Supporting Information, Section S2.2, Figures S6, and S7). Furthermore, three independent ReaxFF-MD metadynamics simulations, of 2.0 ns each, were conducted to explore the selectivity of hydrogenated graphene nanopores concerning a sodium cation (Na^+^). This investigation involved the assessment of the free energy along the collective variable CV1* (see Section 2 Computational Methods for the CV1* definition). The analysis of the sodium cation’s position (see the Supporting Information, Section S2.2, Figure S8) revealed its ability to penetrate through the hydrogenated nanopore with an activation barrier of 33.1 ± 8.8 kJ mol^–1^ (see the Supporting Information, Section S2.2, Figures S9 and S10). These results show that this nanopore size allows an evident and facile flow of water and solvated hydronium ion, as well as the possibility of sodium cation transport, in accordance with previous literature.^62^ Therefore, this graphene nanopore size leads to a low selectivity for proton transport, raising concerns about the possible risk of reactants or other ionic species crossover in proton exchange membrane applications.

### 3.2 Proton Transport from and to a Graphene Nanopore Covalently Functionalized with Ph-SO_3_H or Ph-COOH or Ph-OH in the Aqueous Environment

The effects of various covalent functionalization moieties on the edge of a flexible graphene nanopore were examined in order to achieve a good balance between the selectivity and permeability for this graphene nanopore size. Three systems covalently functionalized at the edge of the previously considered hydrogenated nanopore are investigated and schematically shown in Figure 1d,e,f. Each functionalized system is equilibrated, starting from the anionic moieties: benzenesulfonate, benzoate, or phenolate (see Section 2 for the details). Then, an excess proton has been added to one of the oxygens of each functionalization. After another equilibration run, three independent ReaxFF-MD metadynamics simulations, of 1.0 ns each, were performed to determine the averaged free energy profile along the collective variable CV2 shown in Figure 1d,e,f (see Section 2 Computational Methods for the CV2 definition). The collective variable CV2 is specifically designed to evaluate the free energy profile of the proton traveling from the hydronium in the water bulk to the anionic functional group (Ph-SO_3_^–^, Ph-COO^–^, Ph-O^–^), and *vice versa* from each functional group (Ph-SO_3_H, Ph-COOH, Ph-OH) to the water bulk.

**Figure 2:**
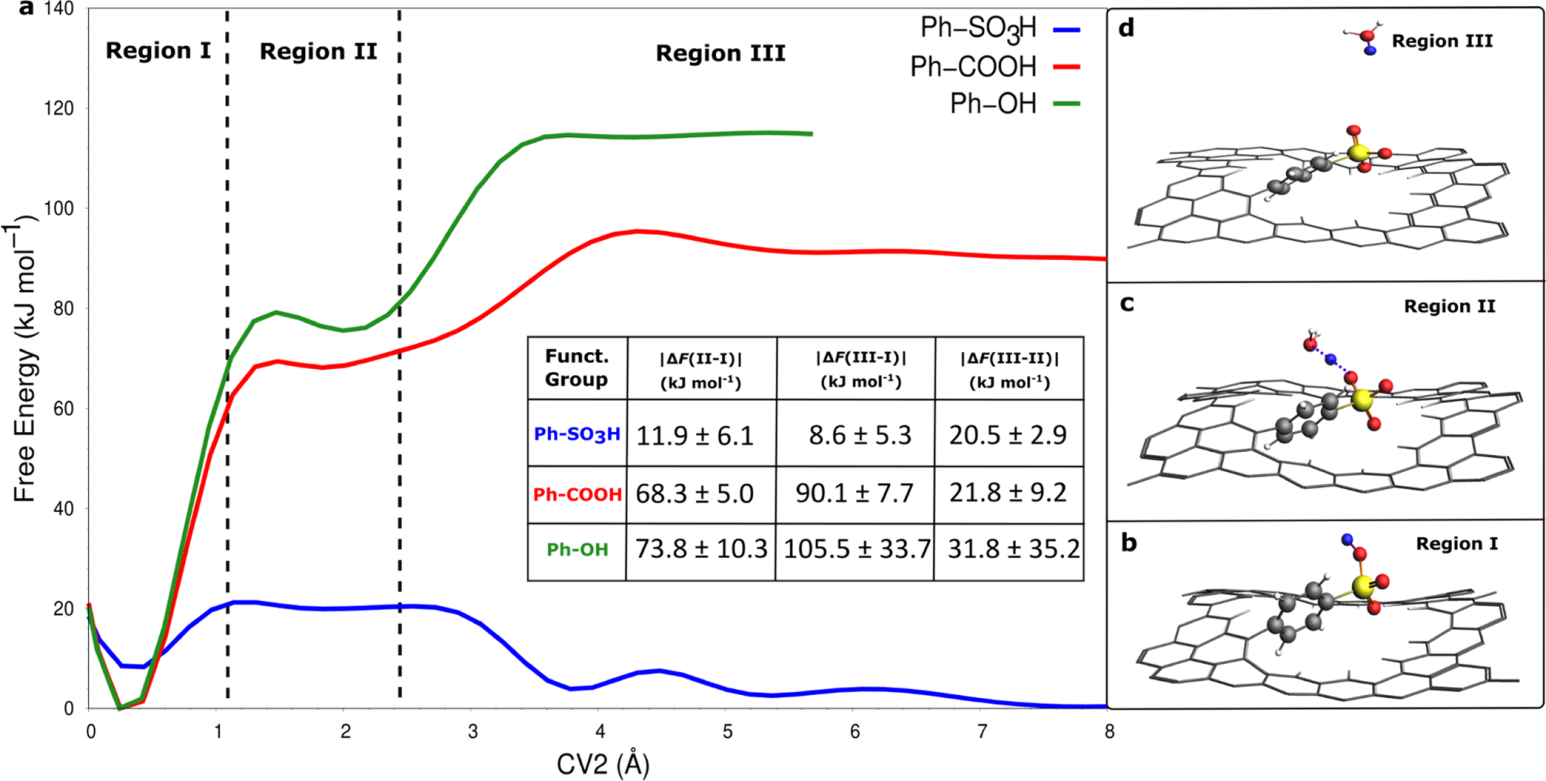
(a) Free energy profiles (kJ mol^–1^) along the collective variable CV2 (Å) averaged over 1.0 ns each of three independent simulations for graphene nanopores functionalized with benzenesulfonic (Ph-SO_3_H, blue line), benzoic (Ph-COOH, red line), and phenolic (Ph-OH, green line) groups, respectively. (b–d) Representative configurations for the benzenesulfonic case corresponding to regions I, II, and III, respectively. The benzenesulfonic functionality and the water molecule involved in the proton hopping are represented by balls and sticks: carbon, oxygen, sulfur, hydrogen, and the excess proton are colored in gray, red, yellow, white, and blue, respectively. The inset table reports the absolute values of the average energy barrier along with corresponding standard deviations of region II (1.1 ≤ CV2 ≤ 2.4 Å) and region III (CV2 ≥ 4.5 Å) with respect to region I (CV2 ≈ 0 Å), and region III (CV2 ≥ 4.5 Å) relative to region II (1.1 ≤ CV2 ≤ 2.4 Å), indicated as |Δ*F* (II–I)|, |Δ*F* (III–I)|, and |Δ*F* (III–II)|, respectively, for each covalently functionalized graphene nanopore system.

During the metadynamics simulations, the proton is indeed observed to hop from the functional group to the water environment and consequently diffuses in the aqueous phase following a characteristic Grotthuss mechanism, exploiting the dynamic hydrogen-bond network.^19,63^ The evolution of CV2 is reported in Figure S11 in the Supporting Information, Section S2.3, for each system and each metadynamics run. From the analysis of the averaged free energy profiles along CV2 shown in Figure 2, three key regions can be distinguished. The first region, termed region I in Figure 2a, corresponds to the free energy around CV2 ≈ 0 Å representing a relatively stable configuration, where the proton is bonded to the functional group, as shown in Figure 2b. Region II in Figure 2a is associated with the sharing of the proton between the oxygen of the functional group and oxygen of a water molecule in the first solvation shell (1.1 ≤ CV2 ≤ 2.4 Å). A representative snapshot of region II is provided in Figure 2c. Region III in Figure 2a corresponds to the proton diffusing in the water environment, as illustrated by a representative snapshot in Figure 2d. The diffusive behavior of the proton in region III is visible in Figure S11 (see the Supporting Information, Section S2.3) as the collective variable CV2 explores a broad range of values (CV2 ≥ 4.5 Å). For the benzenesulfonic-functionalized graphene nanopore, the free energy difference between region III (taken as the minimum of free energy) and region II describes the proton trap step by the benzenesulfonic group. It represents the largest activation barrier for a benzenesulfonic-functionalized system and thus leads to the rate-limiting step for the entire proton transport process |Δ*F* (III–II)| = 20.5 ± 2.9 kJ mol^–1^, as shown in Figure 2a. The proton captured from the water bulk by the benzenesulfonic group will be released into the water bulk overcoming an activation barrier described by the difference in free energy between region II and region I, |Δ*F* (II–I)| = 11.9 ± 6.1 kJ mol^–1^, as shown in Figure 2a. For the benzoic- and phenolic-functionalized systems, the free energy differences between region II and region I (taken as the minimum of free energy) are |Δ*F* (II–I)| = 68.3 ± 5.0 kJ mol^–1^ and |Δ*F* (II–I)| = 73.8 ± 10.3 kJ mol^–1^, respectively. These differences represent the largest activation barriers for both systems and thus lead to the rate-limiting step for proton release into bulk water (Figure 2a). The free energy profiles in region III for the benzoic- and phenolic-functionalized systems are affected by scarce sampling (see Figure 2a green line and the Supporting Information, Section S2.3, Figures S11 and S12), causing a larger statistical error (see |Δ*F* (III–I)| and |Δ*F* (III–II)| in Figure 2 inset table) compared to the values in region I and region II. Nevertheless, the free energy difference between region II and region I, within the statistical error, remains the rate-limiting step for proton release into the bulk water in both the benzoic- and phenolic-functionalized systems. The height of the energy barrier between region II and region I can, to some extent, be correlated to the proton affinities of the functional groups. The proton affinity decreases in the following order from the phenol to benzoic, to benzenesulfonic, according to experimental p*K*_a_ and DFT calculations (see the Supporting Information, Section S2.4, Table S1). The lowest proton affinity of the benzenesulfonic group is indeed reflected in the lower free energy barrier observed between region II and region I (Figure 2a) compared with the other two systems. Furthermore, for the benzenesulfonic-functionalized system, in accordance with the acidic p*K*_a_ value and DFT data (Table S1), the most stable configuration is found in region III when the proton is solvated in water. Conversely, for the benzoic- and phenolic-functionalized graphene nanopore systems, the most stable configurations occur when the proton is bonded to the functional group, as confirmed by the p*K*_a_ values and DFT data (Table S1). From the results in Figure 2, it can be concluded that the proton permeability across the functionalized graphene nanopore is likely higher for the benzenesulfonic system, primarily because of the lower rate-limiting free energy barrier when compared with the benzoic and phenol cases. This phenomenon is associated with a specific type of proton-shuttling mechanism, where the benzenesulfonic functional group acts as a temporary proton trap after the proton has been caught from the water bulk, effectively overcoming the rate-limiting activation barrier between region III and region II.

Based on the previous results, the focus is placed on the benzenesulfonic covalent functionalized system, as this is deemed the most promising in terms of proton permeability. To comprehensively evaluate the energetics and mechanism of the proton transport, the dynamics of the functional group while it is crossing the graphene nanopore is studied when the proton is bonded to it, i.e., when the system is in one relative minimum (proton trap) of the free energy profile shown in Figure 2a,b (region I). Following the same previous computational protocol, three independent ReaxFF-MD metadynamics simulations, of 2.0 ns each, were carried out to estimate the free energy along the collective variable CV3, as schematically described in the Supporting Information (Section S2.3, Figure S13a) (see Section 2 Computational Methods for the CV3 definition). The free energy profile in Supporting Information Section S2.3, Figure S13b shows a minimum of 0.6 ± 0.2 kJ mol^–1^ at CV3 ≈ 0 Å (see the Supporting Information, Section S2.3, Figures S14, and S15). The free energy exhibits an asymmetrical pattern caused by the insufficient sampling of the CV3 positive values, in contrast to the negative ones. Within the range of −2 ≤ CV3 ≤ 2 Å, the free energy increases by approximately 5 kJ mol^–1^, which is only twice the average thermal energy (2*k*_B_*T*_300 K_ ≈ 4.958 kJ mol^–1^), indicating that this functional group can move easily in and out of the nanopore on both sides of the graphene layer. Moreover, the free energy change within the range −2 ≤ CV3 ≤ 2 Å is significantly lower than that associated with the proton trap (between region III and region II in Figure 2a). Therefore, the proton trapping process from the bulk water (Figure 2a) is confirmed as the rate-limiting step for proton transport.

### 3.3 Benzenesulfonic Functional Group as a Shuttle in the Proton Transport Process: Energetics and Dynamics

**Figure 3:**
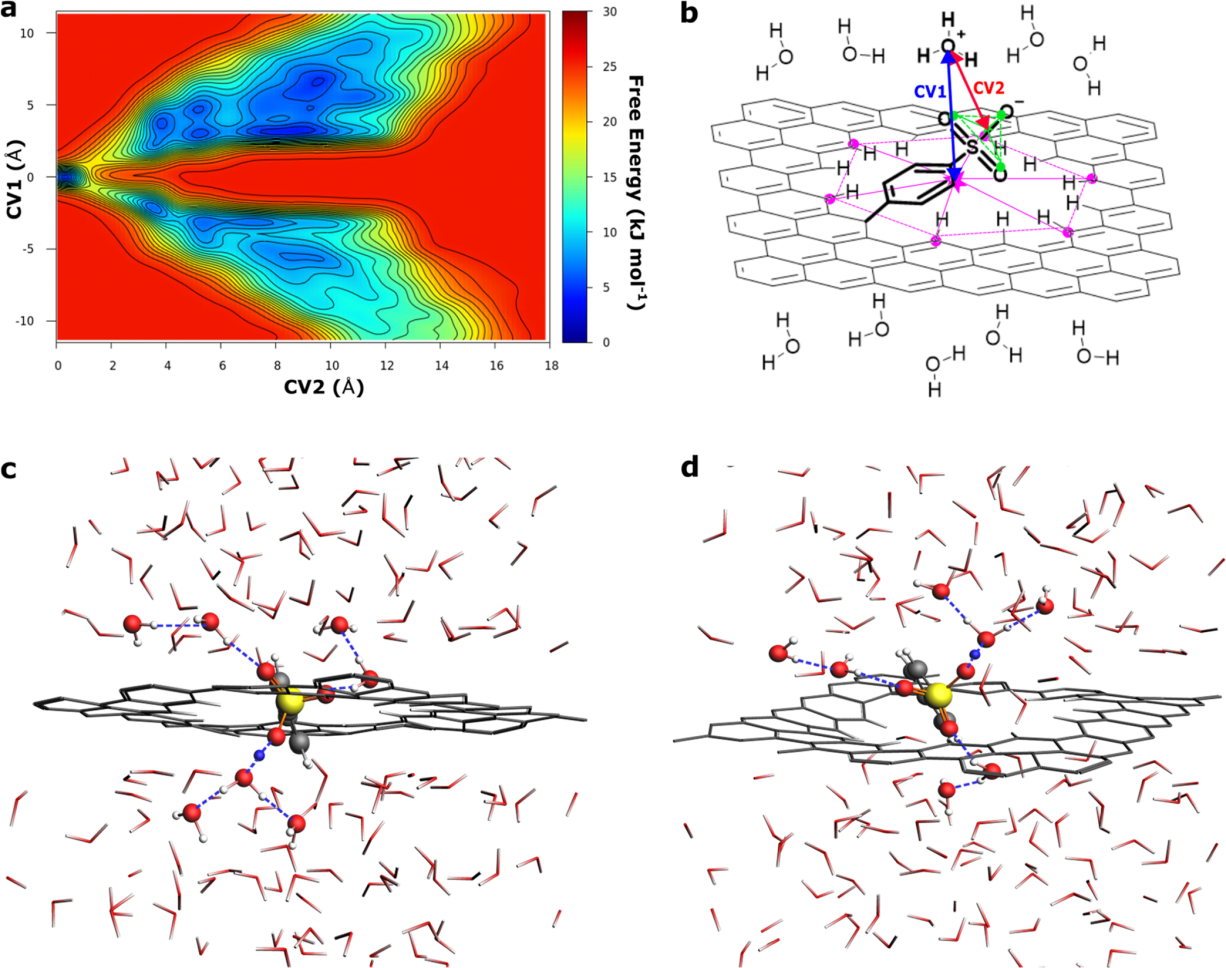
(a) Free energy profiles (kJ mol^–1^) along the collective variables CV1 (Å) and CV2 (Å) averaged over 1.0 ns each of the three final independent simulations for graphene nanopore functionalized with a benzenesulfonic (Ph-SO_3_H) group. (b) Schematic representation of the graphene nanopore covalently functionalized with the benzenesulfonic (Ph-SO_3_H) group and the collective variables CV1 and CV2. CV1 in blue is defined as the distance between the oxygen atom of the hydronium ion and the center of mass of the graphene nanopore along the *z*-axis. The center of mass is calculated using the position of the carbon atoms highlighted in purple. The collective variable CV2, in red, is designed to describe the proton traveling from the hydronium in the water bulk to center of mass of the oxygens (in green) of each anionic functional group (Ph-SO_3_^–^, Ph-COO^–^, Ph-O^–^), and vice versa from the oxygens (in green) of each functional group (Ph-SO_3_H, Ph-COOH, Ph-OH) to the water bulk. (c, d) The representative snapshots along the metadynamics simulation illustrating the proton shuttle mechanism: the proton is above and below the nanopore (CV1 ≈ ±1.0 Å) and is shared between the oxygen of the functional group and oxygen of a water molecule in the first solvation shell (1.1 ≤ CV2 ≤ 2.4 Å). The blue dashed line highlights the hydrogen-bond Grotthuss network (donor–acceptor average distance ≈2.7 Å) connecting the hydronium and water molecules from one side to the other of the graphene nanopore. For clarity, molecules in the network are represented by balls and sticks: carbon, oxygen, sulfur, hydrogen, and the excess protons are colored in gray, red, yellow, white, and blue, respectively. The rest of the water molecules and the graphene sheet are shown as sticks.

The core of this work involves examining the dynamic features of the benzenesulfonic group during proton transport through the graphene nanopore. The benzenesulfonic group enables the opening of a selective proton channel from the water bulk through the graphene nanopore, as illustrated in Figure 3. The benzenesulfonic group can catch the proton within the water layer and subsequently transport it selectively through the nanopore, releasing it to the other side, overall acting like a proton shuttle and initiator of a hydrogen-bond wire through the nanopore. To delve into the energetic and dynamic aspects of the proton shuttling by the benzenesulfonic group, three additional independent ReaxFF-MD metadynamics simulations, of 1.0 ns each, have been performed to determine the free energy profile along the two collective variables CV1 and CV2. In Figure 3a, the 2D plot of the averaged free energy profile for the benzenesulfonic-functionalized graphene nanopore along CV1 and CV2 (see Figure 3b) is presented (see also the Supporting Information, Section S2.5, Figures S16–S19). When CV1 ≥ ±3.0 and CV2 ≥ 4.0 Å, the proton diffuses in the water bulk with a corresponding free energy minimum surface. Interestingly, at CV1 ≈ ±1.0 and 1.1 ≤ CV2 ≤ 2.4 Å, two symmetric channels have been observed with an averaged energy barrier of 14.2 ± 1.9 kJ mol^–1^ relative to the minimum (see Figure 3). This represents the activation energy barrier for the proton trapping on the benzenesulfonic group and closely corresponds within statistical error to the barrier between region III and region II in Figure 2a. Upon surpassing this energy barrier, with both CV1 and CV2 approximately equal to 0 Å, the proton bonds to the benzenesulfonic functionality, leading to a local minimum associated with the proton trap defined in Figures 3 and 2b. For the release of the proton on the opposite side of the graphene nanopore, an energy barrier of 7.1 ± 4.3 kJ mol^–1^ must be overcome (at CV1 ≈ ±1.0 and 1.1 ≤ CV2 ≤ 2.4 Å). Consequently, the benzenesulfonic functionalization opens a proton channel toward the bulk water with the major activation barrier of 14.2 ± 1.9 kJ mol^–1^. This occurs via a dynamical cooperative mechanism that efficiently shuttles the proton across the graphene nanopore. Figure 3c,d shows selected snapshots along the metadynamics trajectory, illustrating this proton shuttle mechanism. As a result, the utilization of benzenesulfonic-functionalized nanoporous graphene in conjunction with Nafion within water–graphene–Nafion systems is proposed^8,18,43^ (10.48550/arXiv.2308.16112). The benzenesulfonic graphene nanopores have the potential to bridge the water environment to the Nafion polymer, creating and aligning selective proton channels between water and Nafion.^8^ This setup would help prevent the crossover of reactants^42,64,65^ and thereby optimize the proton transport within a functionalized graphene–Nafion type PEM.

### 3.4 Proton and Sodium Cation Selectivity for Graphene Nanopore Covalently Functionalized with Ph-SO_3_H or Ph-COOH or Ph-OH in the Aqueous Environment

**Figure 4:**
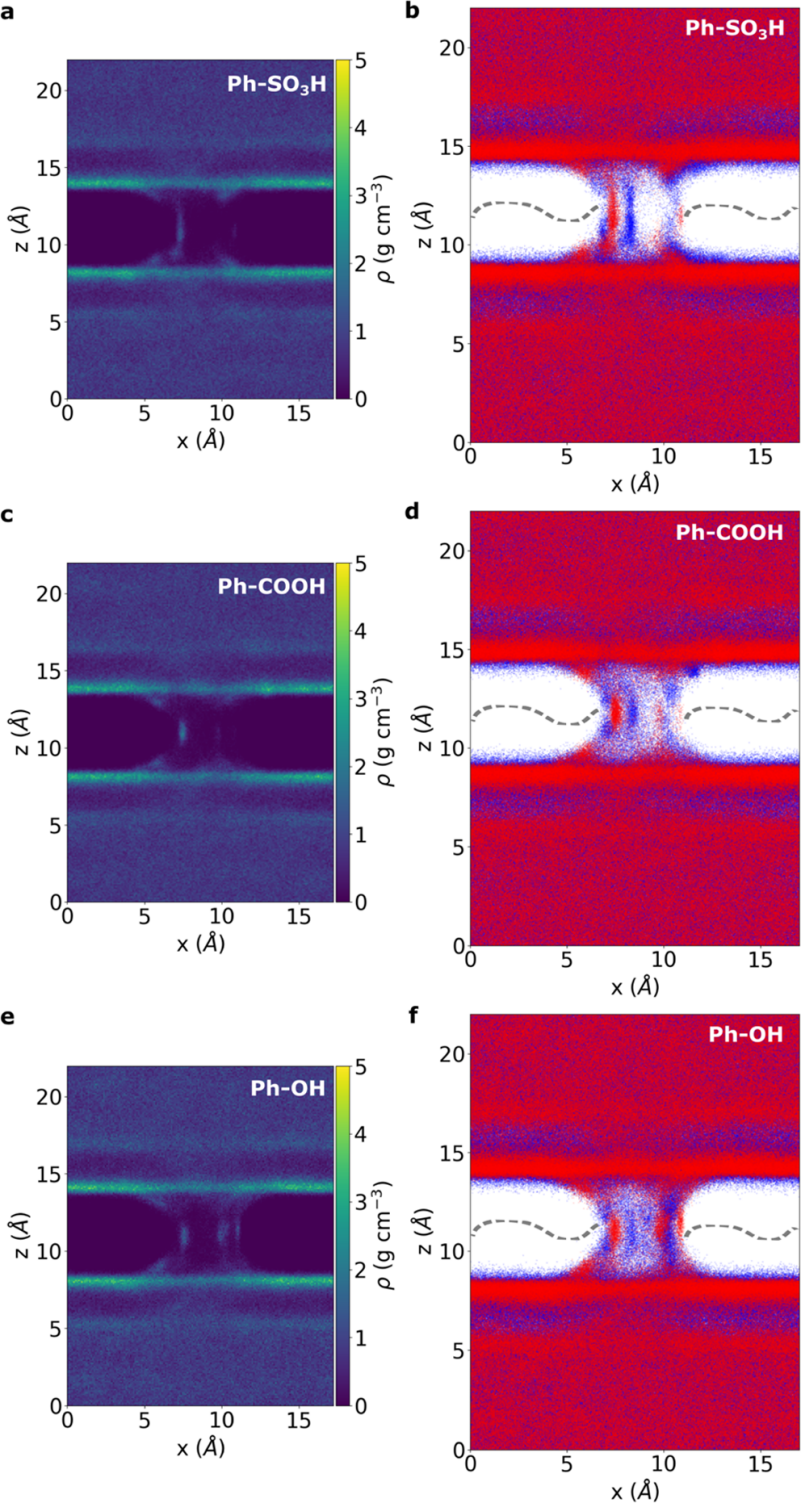
Time-averaged water density extracted from one representative ReaxFF-MD metadynamics simulation for the (a) benzenesulfonic (Ph-SO_3_H), (c) benzoic (Ph-COOH), and (e) phenol (Ph-OH) covalently functionalized graphene nanopore systems in the *x*–*z* plane, averaged along the *y*-axis. The thickness of the void zone above and below the pristine part of the graphene sheet can be identified from the dark area. Projection on the *x*–*z* plane of all oxygen (red) and hydrogen (blue) atom positions for each time step along the same one representative MD simulation trajectory for the (b) benzenesulfonic (Ph-SO_3_H), (d) benzoic (Ph-COOH), and (f) phenol (Ph-OH) covalently functionalized graphene nanopore systems. The graphene layer is represented schematically with a gray dashed line in plots b, d, and f, by the way of illustration.

The water density for each covalently functionalized system has been analyzed as shown in Figure 4a,c,e. Similarly, to the hydrogenated system, a void zone with a thickness of ∼5 Å is visible around the pristine part of the graphene (see the Supporting Information, Section S2.6, Figure S20); however, the water density inside each functionalized graphene nanopore region is different. In Figure 4b,d,f, the positions of the hydrogen and oxygen atoms are shown for each system. The benzenesulfonic-functionalized system in Figure 4a,b appears to have a reduced water content accessing the nanopore compared to the other cases, suggesting a decreased transport of the solvated hydronium ion. To quantify this observation, the water density has been computed consistently with the previous calculation for the hydrogenated case. It is found that only 2.6, 4.0, and 6.7% of the water molecules access the nanopore region for benzenesulfonic, benzoic, and phenol covalent-functionalized systems, respectively (see the Supporting Information, Section S1.2 for more details and Section S2.6, Figure S20). The lowest value for the benzenesulfonic case can be attributed to the increased steric hindrance and therefore a smaller open pore area.^13,62^ Furthermore, three independent ReaxFF-MD metadynamics simulations of 2.0 ns each were conducted to explore the selectivity of the benzenesulfonic-functionalized graphene nanopore concerning a sodium cation (Na^+^). This investigation involved the assessment of the free energy along the collective variable CV1* (see Section 2 Computational Methods for the CV1* definition). The analysis of the sodium cation’s position revealed its inability to traverse the benzenesulfonic-functionalized nanopore (see the Supporting Information, Section S2.6, Figure S21). This analysis confirms the role of benzenesulfonic functionalization in selectively enabling the transport of protons over a cation, such as Na^+^. This proton selectivity arises from the benzenesulfonic steric hindrance, differences in cation size, and the peculiar proton-shuttling mechanism provided by the benzenesulfonic functionalization.

The benzenesulfonic functionalization enhances the proton permeability through the graphene nanopore by lowering the activation barrier compared to the other functional groups and via a characteristic proton shuttling dynamic. Moreover, the benzenesulfonic system increases the selectivity for proton transfer over the sodium cation compared to that of the solely hydrogenated nanopore. Presumably, this system also inhibits the passage of other ionic or molecular species that are computationally detected in smaller not covalently functionalized pores.^16,17,66^ In combination with Nafion, this system could also avoid the undesired crossover of reactants occurring in such sulfonated polymers.^42,67^

### 3.5 Proton Diffusion Coefficient

From the extracted free energy barriers, it is possible to estimate the proton diffusion coefficient *D*_H^+^_.^16,68^ According to the random-walk view of diffusion, the proton diffusion coefficient *D*_H^+^_ can be approximated using the Einstein–Smoluchowski equation^16,68^
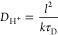
where *l* is the mean step distance for proton transport, taken as 5 Å, which is the distance between the bulk water phases at both sides of the graphene, and *k* is a constant depending on the dimensionality of the random walk. The value *k* = 6 was selected, considering a three-dimensional walk. The τ_D_ is the average time required for a successful proton transport event through the graphene nanopore, which can be calculated as
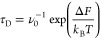
where ν_0_ is the thermal frequency ν_0_ = *k*_B_*T*/*h* with *h* being the Planck constant, *k*_B_ is the Boltzmann constant, *T* is the temperature set at 300 K, and Δ*F* represents the Gibbs free energy for proton diffusion. Assuming negligible volume changes during simulations, the Gibbs free energy can be replaced with the Helmholtz free energy described by the NVT ensemble. For the hydrogenated case, this Δ*F* was found to be 22.3 ± 2.5 kJ mol^–1^ for the flexible case and 11.6 ± 1.1 kJ mol^–1^ for the rigid case (see Figure S3). The Δ*F* value used for the benzenesulfonic-functionalized system is the value extracted from the average height of the barrier at CV1 ≈ ±1.0 and 1.1 ≤ CV2 ≤ 2.4 Å relative to the minimum (see Figure 3a), Δ*F* = 14.2 ± 1.9 kJ mol^–1^, which represents the rate-limiting step for proton transport. The proton diffusion coefficients calculated from the corresponding average Δ*F* values for the flexible hydrogenated and the rigid hydrogenated nanopore systems are *D*_H^+^_ = 3.41 × 10^–11^ m^2^ s^–1^ and *D*_H^+^_ = 2.49 × 10^–9^ m^2^ s^–1^, respectively. However, this higher proton diffusion coefficient compared to that of standard PEM (i.e., Nafion) is not accompanied by a suitable selectivity concerning a sodium cation (Na^+^). On the other hand, the estimated proton diffusion coefficient from the corresponding average Δ*F* value for the benzenesulfonic-functionalized graphene nanopore is *D*_H^+^_ = 8.78 × 10^–10^ m^2^ s^–1^. The benzenesulfonic-functionalized graphene nanopore therefore exhibits a comparable or higher proton diffusion coefficient when compared to both hydrogenated graphene nanopores and experimental and theoretical values for the Nafion system (0.01 × 10^–10^ ≤ *D*_H^+^_ ≤ 5 × 10^–10^ m^2^ s^–1^).^69−72^ Additionally, it demonstrates significant proton selectivity over sodium cations compared to that in the hydrogenated case. These characteristics are essential for optimizing the design of a proton exchange membrane.

## 4 Conclusions

We presented a computational study of proton transport through hydrogenated and three different covalently functionalized graphene nanopore systems. Functionalizing nanoporous graphene with benzenesulfonic groups has shown great promise in developing innovative carbon-based proton exchange membranes (PEMs), owing to its exceptionally low free energy barrier for proton permeability (14.2 ± 1.9 kJ mol^–1^, equivalent to ≈0.15 eV) and outstanding proton selectivity over sodium cations. The present results support the following mechanism for proton transport: the benzenesulfonic functionality captures the proton from the surrounding water and subsequently acts as a temporary proton trap and proton shuttle. The dynamics of the benzenesulfonic moiety facilitates the proton transfer through the nanopore by forming a hydrogen-bond wire connecting the water molecules from both sides of the graphene layer. The rate-limiting step in this process is linked to proton trapping from the water bulk by the benzenesulfonic functionality for this kind of graphene nanopore. The high permeability is the result of a relatively low activation energy barrier ≈0.15 eV for this rate-limiting step. This mechanism yields an attractive proton diffusion coefficient *D*_H^+^_ = 8.78 × 10^–10^ m^2^ s^–1^, comparable to or higher than experimental and theoretical values for the broadly used PEM Nafion (0.01 × 10^–10^ ≤ *D*_H^+^_ ≤ 5 × 10^–10^ m^2^ s^–1^).^69−72^

A versatile role of the benzenesulfonic-functionalized nanopores graphene can therefore be foreseen, either in combination with a sulfonate-based polymer carrier or as a standalone PEM. The proton transport from water bulk to Nafion, mediated by a functionalized benzenesulfonic nanoporous graphene, could achieve more efficient proton permeability and selectivity concerning the standard Nafion case. This potential improvement may address the issue of reactant crossover associated with Nafion, all while retaining the advantage of a high proton diffusion coefficient. Hence, the benzenesulfonic chemical functionalization of *in situ* graphene nanopores represents a new way to enhance the proton permeability and selectivity of nanoporous graphene. In general, the concept of utilizing graphene nanopores, defects, and vacancies for directed functionalization can unlock new opportunities in the production of high-performance and sustainable PEMs. This research may stimulate the design and synthesis of other novel functionalized PEMs with improved selectivity and competitive proton diffusivity, prospecting a “passing the baton” from the current state-of-the-art Nafion-based membranes to a novel class of hybrid 2D PEMs for applications in energy conversion devices.

## Supporting Information Available

Computational methods: further validation of the ReaxFF force field via p*K*_a_ estimation of the benzenesulfonic acid (S1.1); water density ratio in the graphene nanopore relative to the bulk (S1.2); supporting results; free energy profiles along the collective variable CV1 of the flexible and rigid hydrogenated graphene nanopore systems (S2.1); selectivity of proton transport through a hydrogenated graphene nanopore over the sodium cation (S2.2); proton transport from and to a graphene nanopore covalently functionalized with Ph-SO_3_H, Ph-COOH, and Ph-OH, and the aqueous environment (S2.3); DFT estimation of proton affinities (S2.4); benzenesulfonic functional group as a shuttle in the proton transport process: energetic and dynamics (S2.5); proton and sodium cation selectivity for graphene nanopore covalently functionalized with Ph-SO_3_H or Ph-COOH or Ph-OH in the aqueous environment (S2.6) (PDF)
