# Supplementary material for: Covalent Benzenesulfonic Functionalization of a Graphene Nanopore for Enhanced and Selective Proton Transport
Source: J Phys Chem C Nanomater Interfaces. 2024 Feb 21;128(8):3514–24. doi: 10.1021/acs.jpcc.3c07406 (PMC10910585; doi:10.1021/acs.jpcc.3c07406)
Supplement: Supplementary file 1 — jp3c07406_si_001.pdf [file jp3c07406_si_001.pdf]

Covalent Benzenesulfonic Functionalization of a Graphene Nanopore for Enhanced and Selective Proton Transport

Supporting Information

Dario Calvani[1], Bas Kreupeling[1], G. J. Agur Sevink[1], Huub J.M. de Groot[1], Grégory F. Schneider[1*], Francesco Buda[1*]*

[1] Leiden Institute of Chemistry, Leiden University, PO Box 9502, 2300 RA Leiden (The Netherlands)

S1. Supporting Computational Methods

- **S1.1 A further validation of the ReaxFF force field via pK_a estimation of the benzenesulfonic acid**
- **S1.2 Water density ratio in the graphene nanopore relative to the bulk**

S2 Supporting Results

- **S2.1 Free energy profiles along the Collective Variable CV1 of the flexible and rigid hydrogenated graphene nanopore systems**
- **S2.2 Selectivity of proton transport through a hydrogenated graphene nanopore over sodium cation**
- **S2.3 Proton transport from-and-to a graphene nanopore covalently functionalized with Ph-SO_3H, Ph-COOH, and Ph-OH, and the aqueous environment**
- **S2.4 DFT estimation of proton affinities**
- **S2.5 The benzenesulfonic functional group as a shuttle in the proton transport process: energetic and dynamics**
- **S2.6 Proton and sodium cation selectivity for graphene nanopore covalently functionalized with Ph-SO_3H, or Ph-COOH, or Ph-OH, in aqueous environment**

S1. Supporting Computational Methods

S1.1 A further validation of the ReaxFF force field via p*K*a estimation of the benzenesulfonic acid. In this section, we had further validated the ReaxFF CHONSMgPNaTiClFKLi.ff force field[1,2] for describing the benzenesulfonic functionality. This validation was carried out using unbiased molecular dynamics to extrapolate the reaction free energy for the deprotonation process and, subsequently, determine the acidity constant, pK_a.[3,4] For this validation, we employed a simulation box with dimensions of 12.91 Å × 12.91 Å × 12.91 Å, containing a benzenesulfonic acid molecule solvated by 62 water molecules (Figure S1a). Three unbiased ReaxFF-MD NVT equilibrations of 1.5 ns each were conducted using the LAMMPS software,[5] with the CSVR thermostat[6] maintaining a temperature of 300 K. The simulations utilized a time step of 0.25 fs and a damping constant of 100 fs for temperature control. During these simulations, a Collective Variable 4 (CV4) was employed.[4] The CV4 is defined as the distance, $r_{\mathrm{Sp}} = \|\boldsymbol{q}_\mathrm{S} - \boldsymbol{q}_\mathrm{p}\|$, between the hydronium and the sulfur of the benzenesulfonic acid. The $\boldsymbol{q}_\mathrm{S}$ represents the position of the sulfur atom of the benzenesulfonic acid, while $\boldsymbol{q}_\mathrm{p}$, denotes the position of the hydronium ion, computed as a weighted sum over all positions, $\boldsymbol{q}_i$, of oxygen atoms capable of binding the proton:

$$\boldsymbol{q}_\mathrm{p} = \sum_i w_i \boldsymbol{q}_i$$

with the weights,

$$w_i = \frac{\exp[\lambda(n_i - n_{0,i})]}{\sum_i \exp[\lambda(n_i - n_{0,i})]}$$

The position $\boldsymbol{q}_\mathrm{p}$ is thus obtained as an exponentially weighted average of all proton acceptor positions, which are the water and oxygen atoms of the benzenesulfonic acid. With a positive number for the parameter λ, the weighting selects the oxygen(s) with highest coordination number with respect to the H-atoms, n_{OH}. Here $n_{0,i}$ is the default number of hydrogens bonded to atom *i* (without the proton), which is 2 for a water oxygen and 0 for the benzenesulfonic acid oxygen atoms. The coordination number $n_{\mathrm{OH}} = n_i$ is computed with:

$$n_{xy} = \sum_{i=1}^{N_x} \sum_{j=1}^{N_y} \frac{1 - \left(\frac{r_{ij}}{r_0}\right)^n}{1 - \left(\frac{r_{ij}}{r_0}\right)^m}$$

where $x = \mathrm{O}$ and $y = \mathrm{H}$.

The CV4 is not highly sensitive to the exact values of the switching function, and the same parameters of the switching function are effective across the entire pK_a spectrum. The following parameters values were used: $n = 12$, $m = 24$, $\lambda = 8$, and $r_0 = 1.3$ Å.

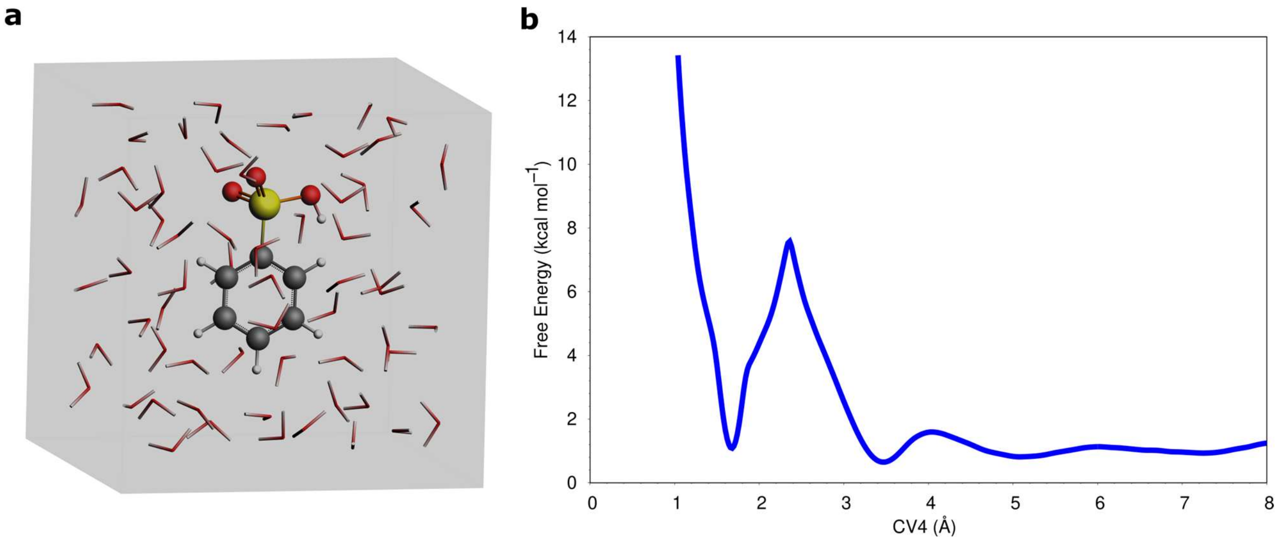

Figure S1. **a**, Representative configuration for the benzenesulfonic acid solvated in water. The benzenesulfonic acid is represented by balls and sticks: carbon, oxygen, sulfur, and hydrogen are colored in grey, red, yellow, and white, respectively; water molecules are represented by sticks. **b**, Free energy profile (kcal mol^{-1}) for proton dissociation reaction along the Collective Variable CV4 (Å) averaged over 1.5 ns each of three independent simulations for benzenesulfonic acid solvated in water.

The estimated averaged free energy profile and evolution of the CV4 are shown in Figure S1b and Figure S2, respectively. The free energy profile along the path shows three states (Figure S1b). The first minimum at 1.7 Å corresponds to the proton bonded to the benzenesulfonic functionality; the top of the energy barrier corresponds to the sharing of the proton with the first water solvation shell, CV4 = 2.38 Å; the second minimum is associated to the proton solvated in the first water hydration shell (contact ion pair),[4] CV4 ≈ 3.4 Å; and finally the proton diffuses in the water bulk CV4 ≥ 4 Å.

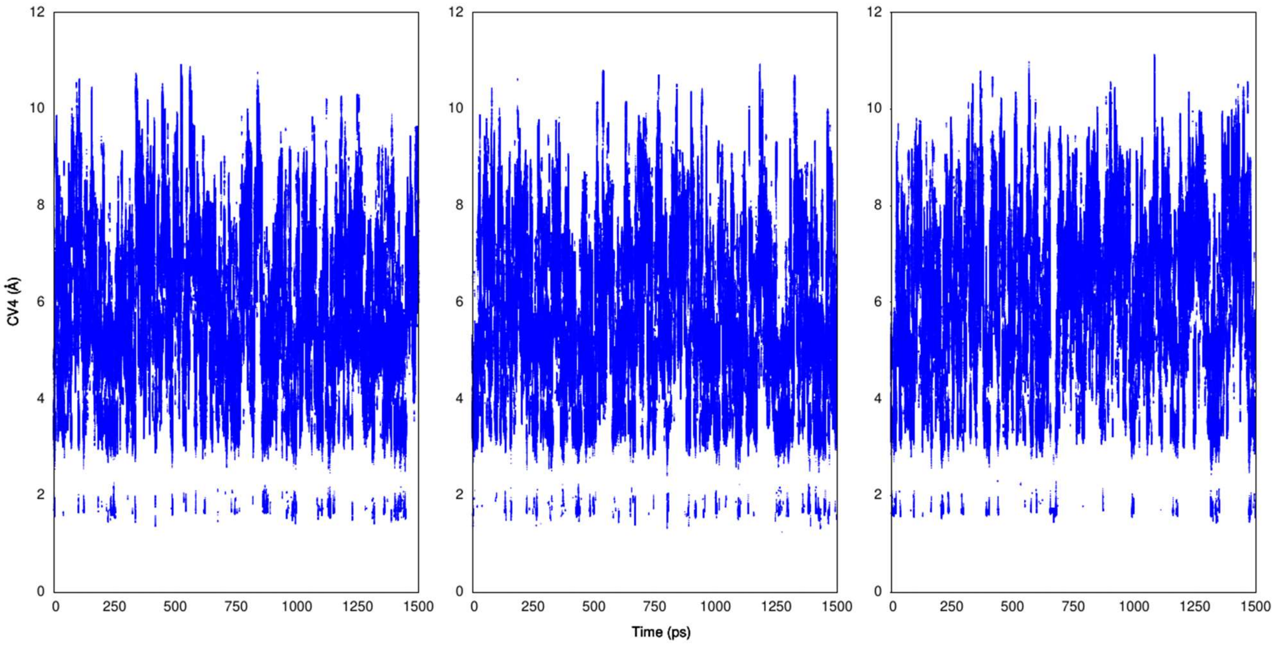

Figure S2. Time evolution of the CV4 (Å) for the simulation of benzenesulfonic acid in water solvation.

The free energy difference between the undissociated acid state (1.1 Å ≤ CV4 ≤ 2.38 Å) and the solvated hydronium ion (2.38 Å ≤ CV4 ≤ 7 Å) is calculated by

$$\Delta F = -k_{\mathrm{B}}T \ln\left(\frac{\int_{2.38}^{7} \exp\left(-F\left(r_{\mathrm{Sp}}\right)/k_{\mathrm{B}}T\right)\, dr_{\mathrm{Sp}}}{\int_{1.1}^{2.38} \exp\left(-F\left(r_{\mathrm{Sp}}\right)/k_{\mathrm{B}}T\right)\, dr_{\mathrm{Sp}}}\right) = -2.08 \text{ kcal mol}^{-1}$$

where $F\left(r_{\mathrm{Sp}}\right)$ is the free energy profile along r_{Sp} = CV4.

The pK_{a} of the benzenesulfonic acid can be obtained from the reaction free energy difference using the following equation:[4]

$$\mathrm{p}K_a = \frac{\Delta F}{k_{\mathrm{B}}T \ln(10)} = -1.52$$

The obtained pK_{a} of −1.52 is in a reasonable agreement with the experimental value of −2.50 reported in the Supporting Information, section S2.4 Table S1.[7] This analysis provided a further validation of the ReaxFF force field CHONSMgPNaTiClFKLi.ff force field[1,2] in order to be used for estimating the energetics and dynamics of a graphene nanopore functionalized with benzenesulfonic group in water environment.

S1.2 Water density ratio in the graphene nanopore relative to the bulk. The water densities for the hydrogenated and covalently functionalized graphene nanopore systems are computed employing the MDAnalysis package.[8,9] The calculation of the water density involved the utilization of the oxygen atom positions from each ReaxFF-MD metadynamics simulation for each of the hydrogenated and functionalized graphene nanopore systems (Figure 4a,c,e). From the water density data, the water density ratios of each system reported in sections 3.1 and 3.4, averaged over the corresponding three ReaxFF-MD metadynamics simulations, were extracted relative to the water bulk value ($\rho_{water} \sim 1.0$ g cm^{-3}) , normalized within a 3 Å thick layer respect to the center of mass of the graphene layer (Figure S7, Figure S20).

S2 Supporting Results

S2.1 Free energy profiles along the Collective Variable CV1 of the flexible and rigid hydrogenated graphene nanopore systems. The free energy associated with proton transport for both a freely moving, *i.e.* flexible, and a constrained, *i.e.* rigid hydrogenated graphene nanopores, was determined to assess the role of graphene sheet undulations. The weak symmetry breaking of the free energy profiles around the nanopore center reflects finite sampling, and it is slightly more prominent when the graphene is flexible. Figure S3 suggests that the motion of the graphene sheet is likely to result in an increase in the energy barrier for proton transport across the hydrogenated graphene nanopore when compared to the rigid case, with an estimated change in free energy of approximately $\Delta\Delta F \approx 10.7 \pm 2.8$ kJ mol^{-1}.

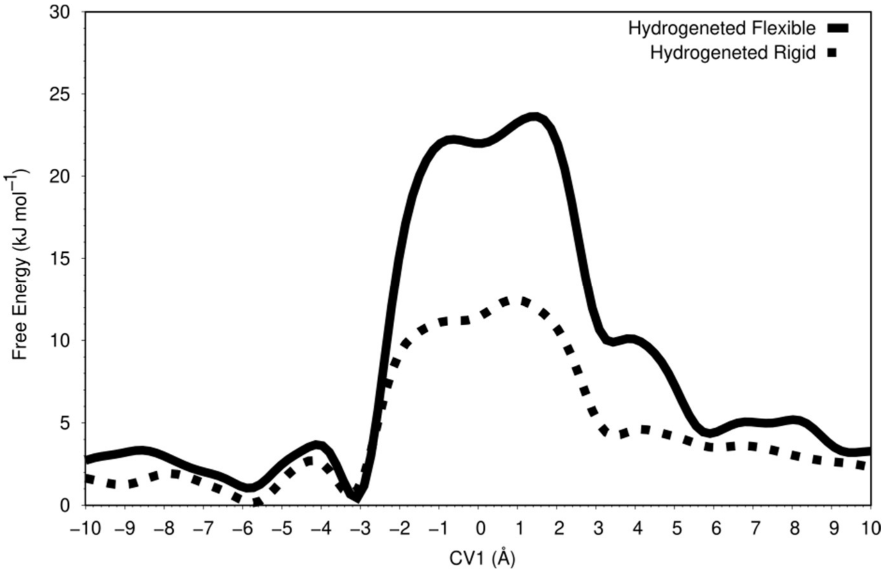

Figure S3. Free energy profiles (kJ mol^{-1}) of proton transport through flexible (solid line) and rigid (dashed line) hydrogenated graphene nanopore systems along the Collective Variable CV1 (Å) averaged over 1.0 ns each of the three final independent simulations.

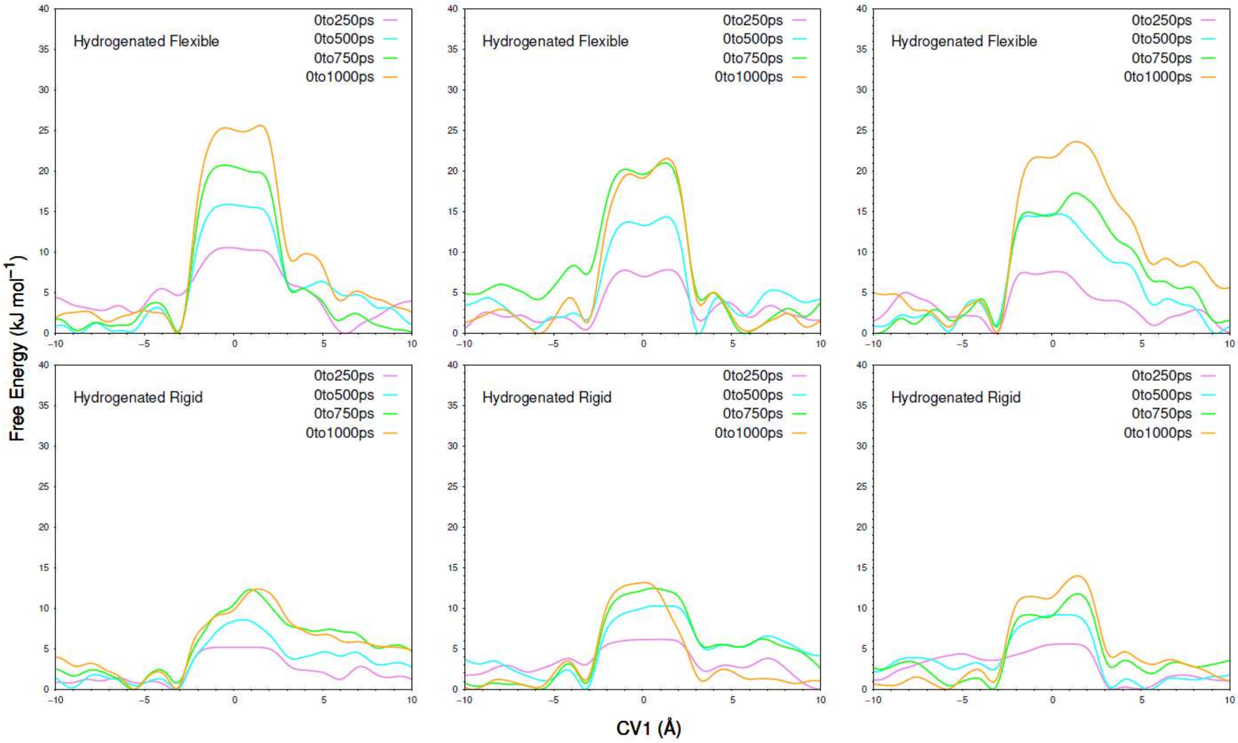

Figure S4. Free energy convergence (kJ mol^{-1}) of proton transport through flexible (top) and rigid (bottom) hydrogenated graphene nanopore systems along CV1 (Å). To assess the convergence of a metadynamics simulations, each free energy profile is extracted after 0.25 ns (violet line), 0.5 ns (cyan line), 0.75 ns (green line) and 1.0 ns (orange line) of each simulation, with a deposition stride every 25 fs, and the global minimum is set to zero in all profiles.

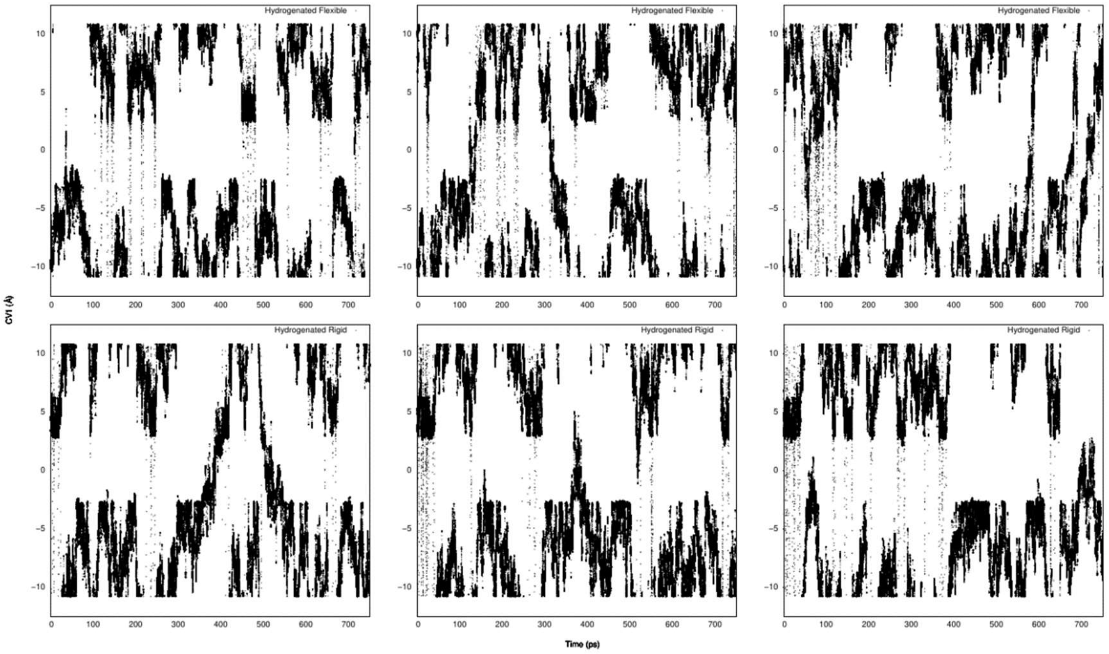

Figure S5. Time evolution of the Collective Variable CV1 (Å) for each of the three final independent simulations of the flexible (top) and rigid (bottom) hydrogenated graphene nanopore systems, respectively.

S2.2 Selectivity of proton transport through a hydrogenated graphene nanopore over sodium cation. To analyze the proton selectivity, the density of the water shell around the graphene nanopore was visualized, see Figure S6a,c. Water is excluded from a ~ 5 Å layer next to the pristine part of the graphene layer, in agreement with previous findings.[10–12] On the other hand, water can penetrate the hydrogenated graphene nanopore as seen in Figure S6b,d showing the atomistic positions of hydrogen and oxygen. From the water density data, the water density ratio reported in section 3.1 were extracted relative to the water bulk value (ρ_{water} ~ 1.0 g cm^{-3}) averaged within a 3 Å thick layer respect to the center of mass of the graphene layer (Figure S7). In Figure S8, Figure S9, and Figure S10 we depict the analysis of the selectivity of the flexible hydrogenated graphene nanopore with respect to a sodium cation (Na^+), as discussed in Section 3.1.

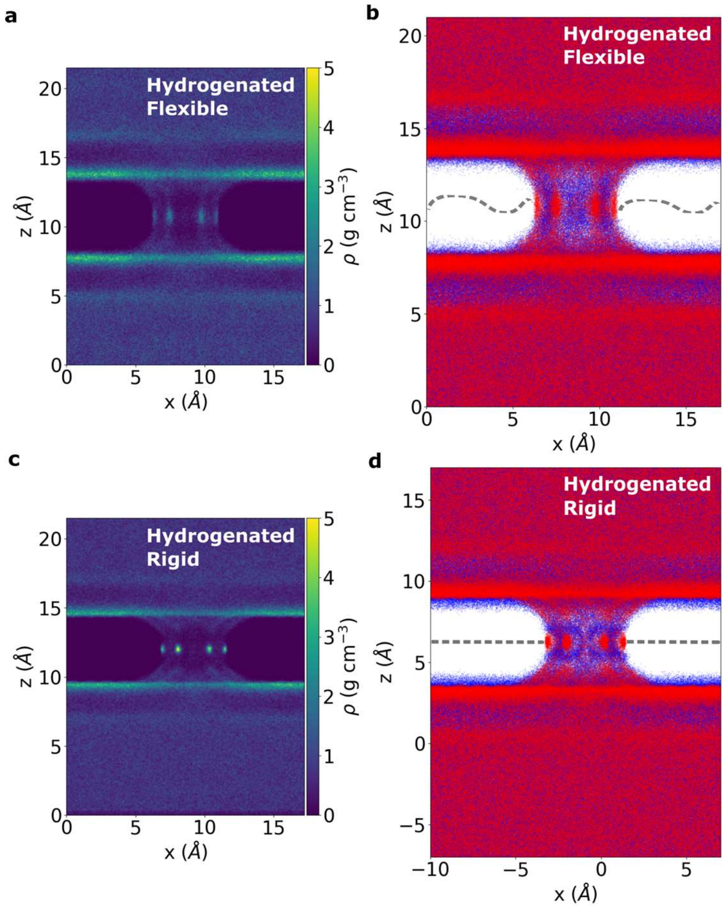

Figure S6. Time-averaged water density extracted from one representative ReaxFF-MD metadynamics simulation for the **a**, flexible and **c**, rigid hydrogenated graphene nanopore systems in the *x*-*z* plane, averaged along the *y*-axis. The thickness of the void zone above and below the pristine part of the graphene sheet can be identified from the dark area. Projection

on the *x-z* plane of all oxygen (red) and hydrogen (blue) atoms positions for each time step along the same one representative MD simulation trajectory for the **b**, flexible and **d**, rigid hydrogenated cases. The graphene layer is represented schematically with a gray dashed line in plots **b**, and **d**, by way of illustration.

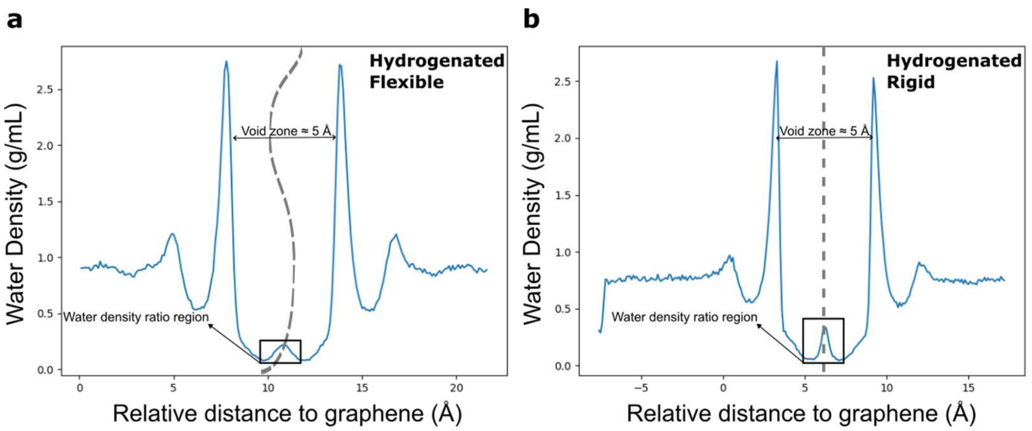

Figure S7. Density profiles (g/mL) of water along the relative distance to the graphene (Å) for the **a**, flexible and **b**, rigid hydrogenated graphene nanopore systems. The void zone of ~ 5 Å and the region for the water density ratio estimations have been highlighted. The graphene layer is represented with a gray dashed line by way of illustration.

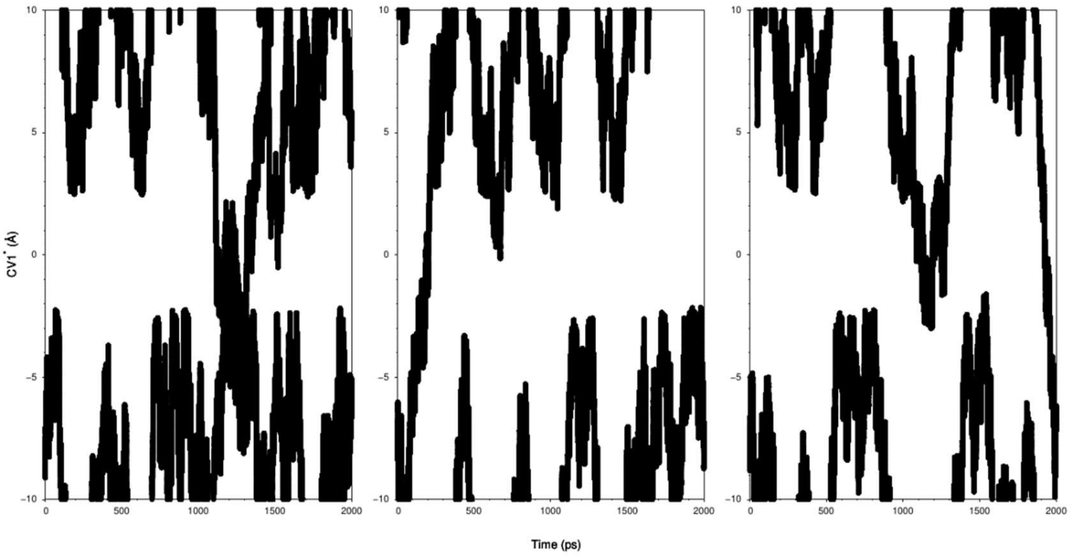

Figure S8. Time evolution of the Collective Variable CV1* (Å) for the three independent simulations of the flexible hydrogenated graphene nanopore system with a sodium cation in the water bulk.

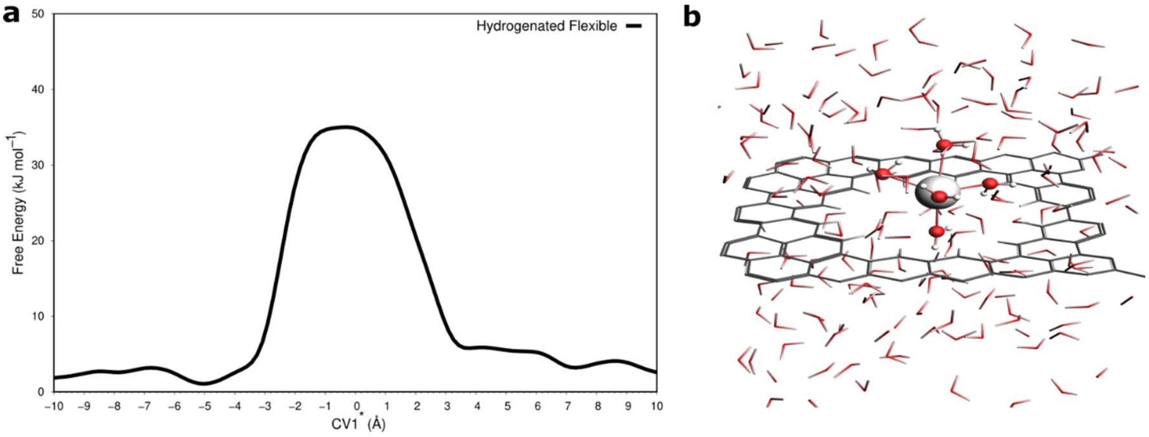

Figure S9. **a**, Free energy profile (kJ mol^{-1}) of the sodium cation transport through a flexible hydrogenated graphene nanopore system along the Collective Variable CV1* (Å) averaged over 2.0 ns each of the three final independent simulations. **b**, Representative configuration for the flexible hydrogenated graphene nanopore system. The sodium cation (Na^+) and the water molecules involved in its first solvation shell are represented by balls and sticks: carbon, oxygen, hydrogen, and the sodium cation are colored in grey, red, white, and light grey, respectively.

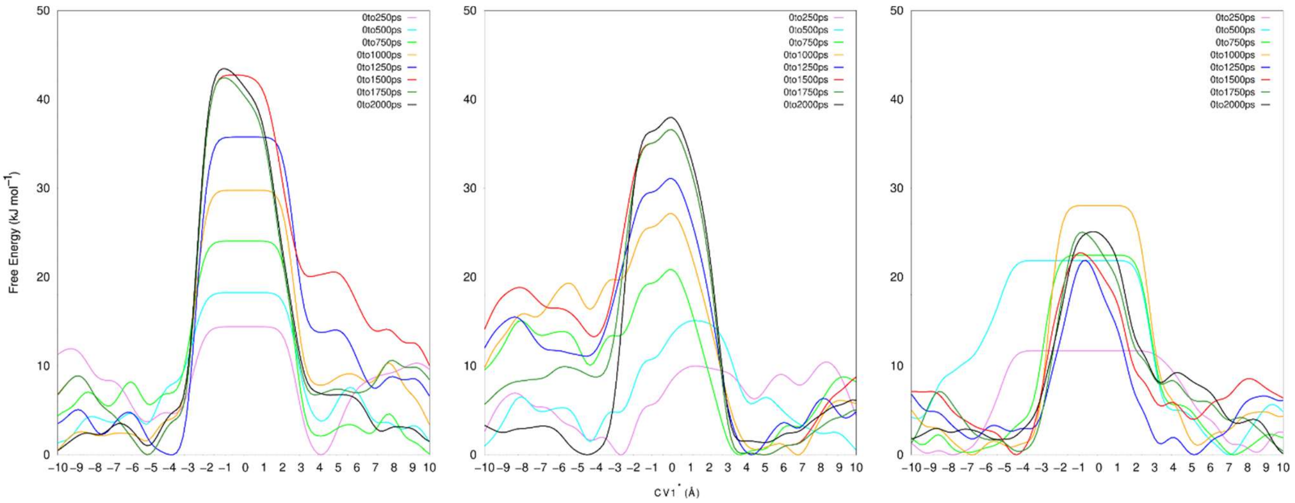

Figure S10. Free energy convergence (kJ mol^{-1}) of sodium cation transport through the flexible hydrogenated graphene nanopore system along CV1* (Å). To assess the convergence of a metadynamics simulations, each free energy profile is extracted after 0.25 ns (violet line), 0.5 ns (cyan line), 0.75 ns (green line), 1.0 ns (orange line), 1.25 ns (blue line), 1.5 ns (red line), 1.75 ns (dark green line) and 2.0 ns (black line) of each simulation, with a deposition stride every 25 fs, and the global minimum is set to zero in all profiles.

S2.3 Proton transport from-and-to a graphene nanopore covalently functionalized with Ph-SO_3H, Ph-COOH, and Ph-OH, and the aqueous environment.

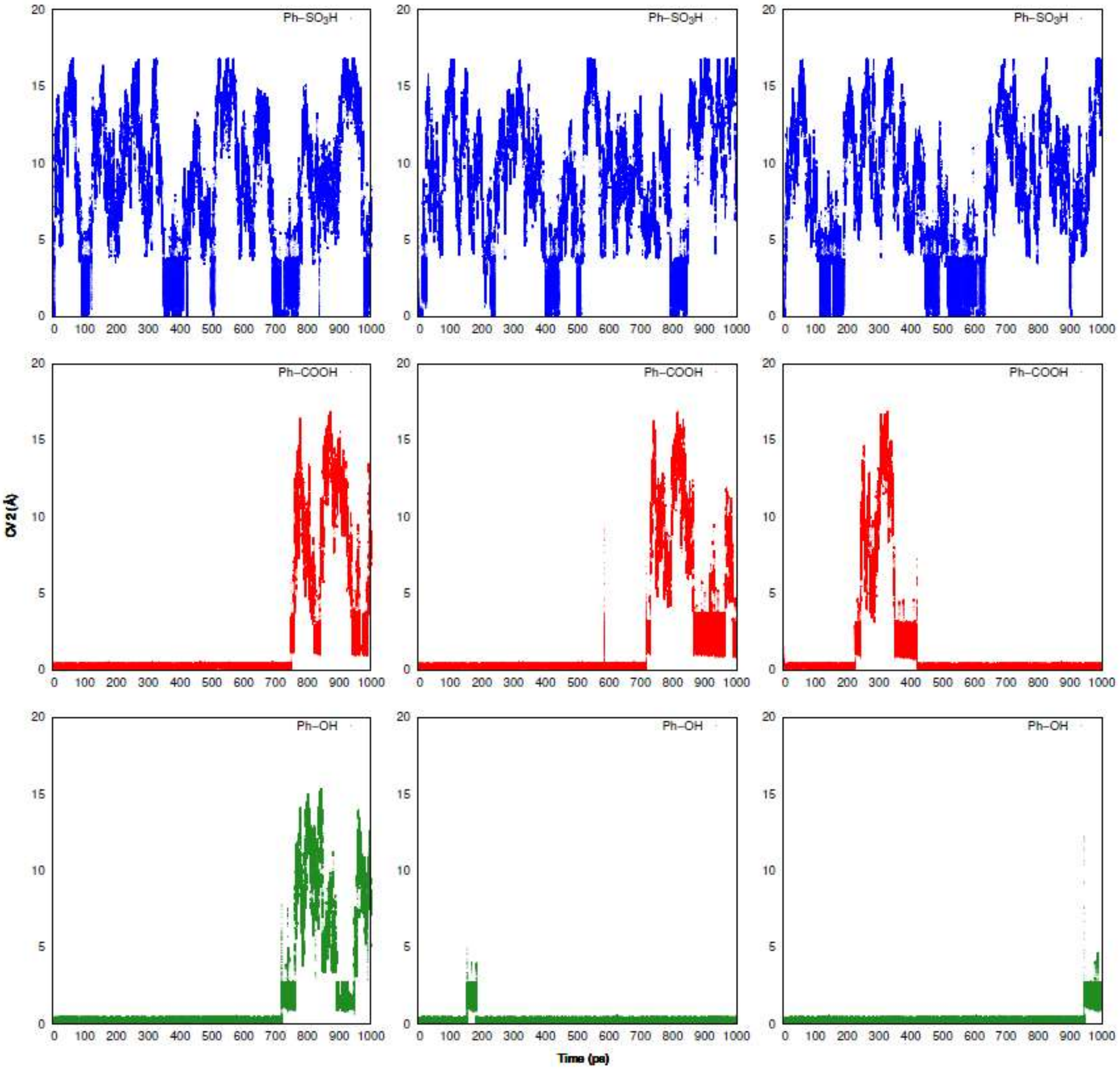

Figure S11. Time evolution of the Collective Variable CV2 (Å) for each of the three final independent simulations of the benzenesulfonic (Ph-SO_3H - blue points), benzoic (Ph-COOH - red points) and phenol (Ph-OH - green points) covalently functionalized graphene nanopore systems, respectively.

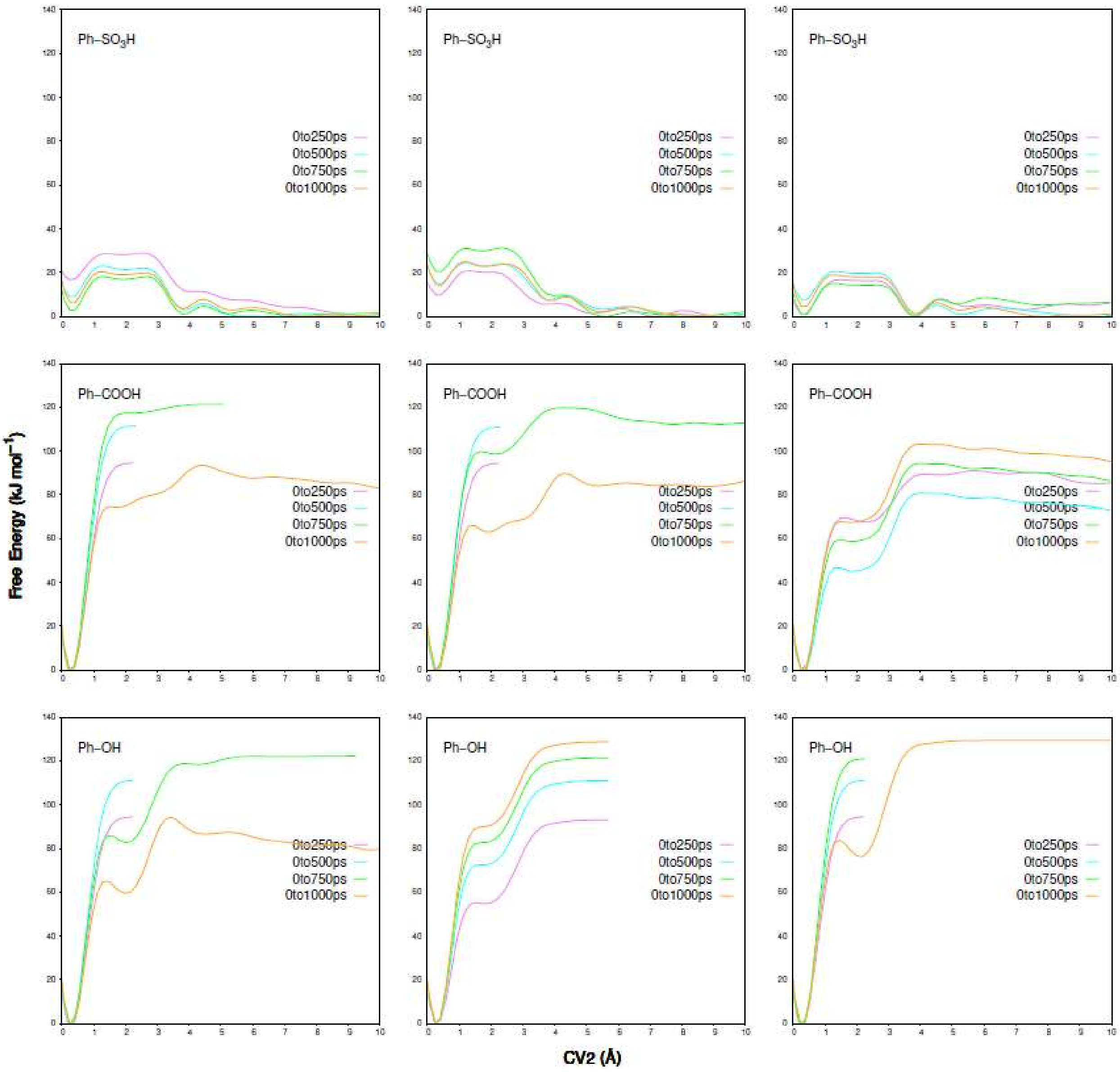

Figure S12. Free energy convergence (kJ mol^{-1}) of proton transport through benzenesulfonic (Ph-SO_3H), benzoic (Ph-COOH), and phenol (Ph-OH) covalently functionalized graphene nanopore systems, respectively, along CV2 (Å). To assess the convergence of a metadynamics simulations, each free energy profile is extracted after 0.25 ns (violet line), 0.5 ns (cyan line), 0.75 ns (green line), and 1.0 ns (orange line) of each simulation, with a deposition stride every 25 fs, and the global minimum is set to zero in all profiles.

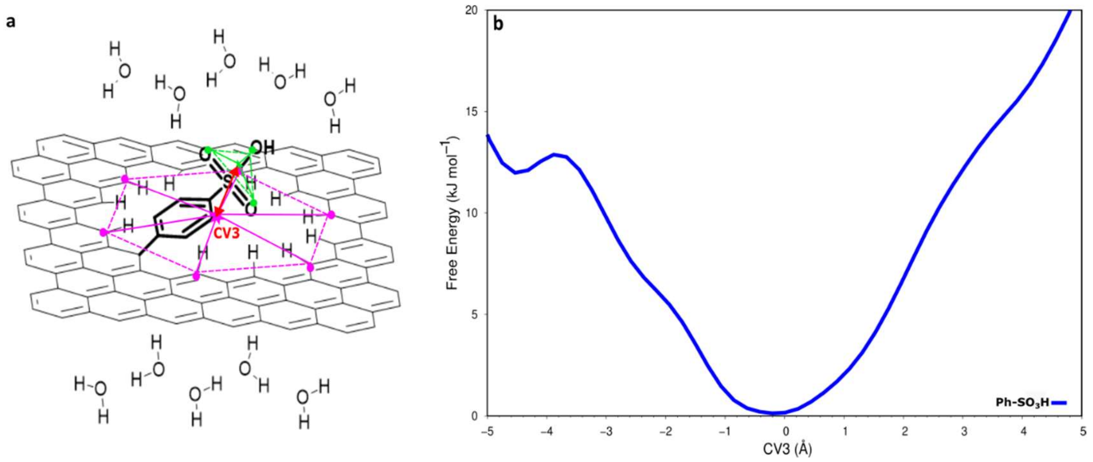

Figure S13. **a**, Schematic representation of the graphene nanopore covalently functionalized with benzenesulfonic and the Collective Variable CV3, in red, defined as the difference between *z*-components of the center of mass of the oxygens (in green) of the benzenesulfonic functionality and the center of mass of the graphene nanopore calculated from the position of the carbon atoms highlighted in purple. **b**, Free energy profile (kJ mol^{-1}) along the Collective Variable CV3 (Å) averaged over 2.0 ns each of the three final independent simulations for the benzenesulfonic (Ph-SO_3H) covalent functionalized graphene nanopore system.

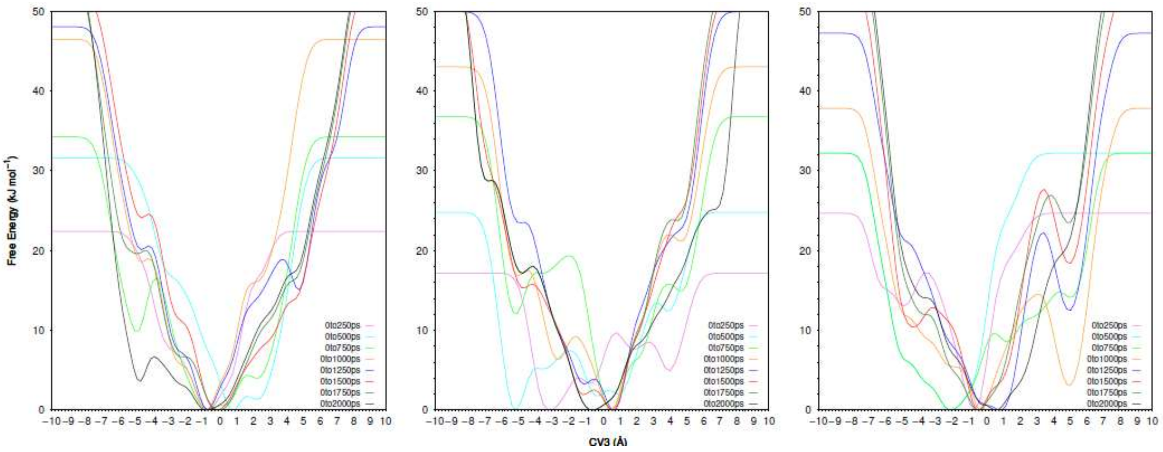

Figure S14. Free energy convergence (kJ mol^{-1}) of proton transport through benzenesulfonic (Ph-SO_3H) covalently functionalized graphene nanopore system along CV3 (Å). To assess the convergence of a metadynamics simulations, each free energy profile is extracted after 0.25 ns (violet line), 0.5 ns (cyan line), 0.75 ns (green line), 1.0 ns (orange line), 1.25 ns (blue line), 1.5 ns (red line), 1.75 ns (dark green line) and 2.0 ns (black line) of each simulation, with a deposition stride every 25 fs, and the global minimum is set to zero in all profiles.

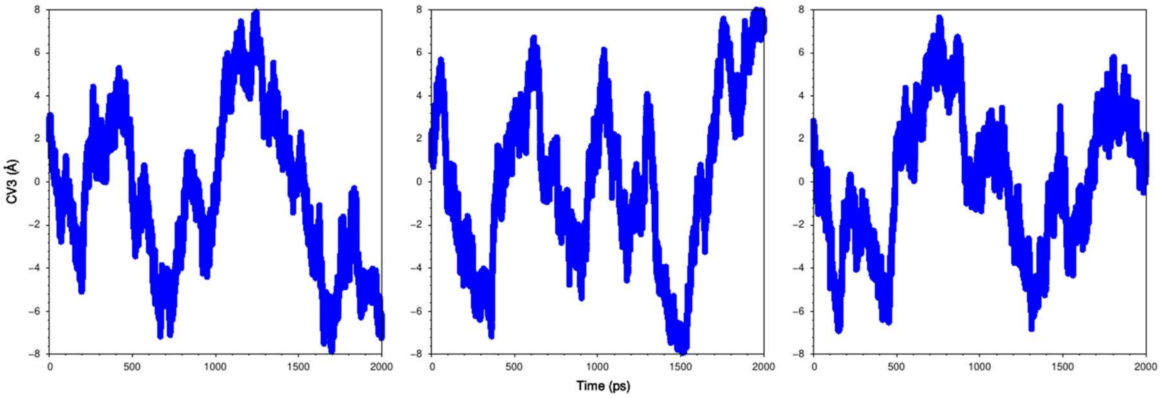

Figure S15. Time evolution of the Collective Variable CV3 (Å) for the three final independent simulations of the benzenesulfonic (Ph-SO_3H - blue points) covalent functionalized graphene nanopore system.

S2.4 DFT estimation of proton affinities. DFT calculations are performed to obtain an overview of the proton affinity (PA) for the three different covalent functionalizations, in vacuum and water continuum model solvent (COSMO), see section 2 Computational Methods for the details. The PAs are determined for the explicative reaction $A^{-} + H^{+} \rightarrow AH$ at temperature of 0 K.[13] It is defined as PA = $E(A^{-}) + E(H^{+}) - E(AH)$ for both cases in vacuum and COSMO, respectively. Only the change in potential energy contribution to the PA is considered. The results reported in Table S1 for the vacuum and COSMO simulations show an increase in affinity from the benzenesulfonic acid to the phenol group. This result is in line with the experimentally determined pK_a.[7]

**Functional Group**	**Vacuum (kJ mol^{-1})**	**COSMO (kJ mol^{-1})**	**Exp. pK_a[7]**
Benzenesulfonic acid	1353.83	615.47	−2.50
Benzoic acid	1459.83	687.54	4.20
Phenol	1495.83	743.90	9.95

Table S1. DFT Proton Affinity estimations (kJ mol^{-1}) for benzenesulfonic acid, benzoic acid, and phenol molecular systems, in vacuum and water continuum model solvent (COSMO), and the corresponding experimental pK_a values.

S2.5 The benzenesulfonic functional group as a shuttle in the proton transport process: energetic and dynamics.

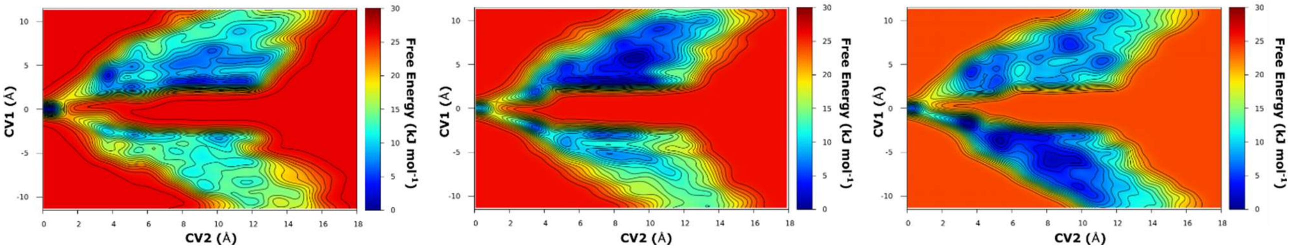

Figure S16. Free energy profiles (kJ mol^{-1}) along the Collective Variable CV1 (Å) and CV2 (Å), over 1.0 ns each, of the three final independent simulations for graphene nanopore functionalized with a benzenesulfonic (Ph-SO_3H) group.

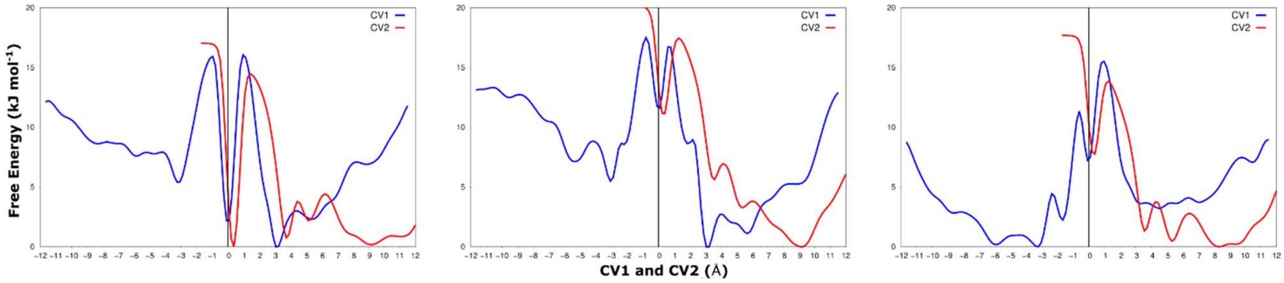

Figure S17. Separate free energy profiles (kJ mol^{-1}) along the Collective Variables CV1 (Å) and CV2 (Å) in blue and red lines, respectively, over 1.0 ns each, of the three final independent simulations of the graphene nanopore functionalized with a benzenesulfonic (Ph-SO_3H) group.

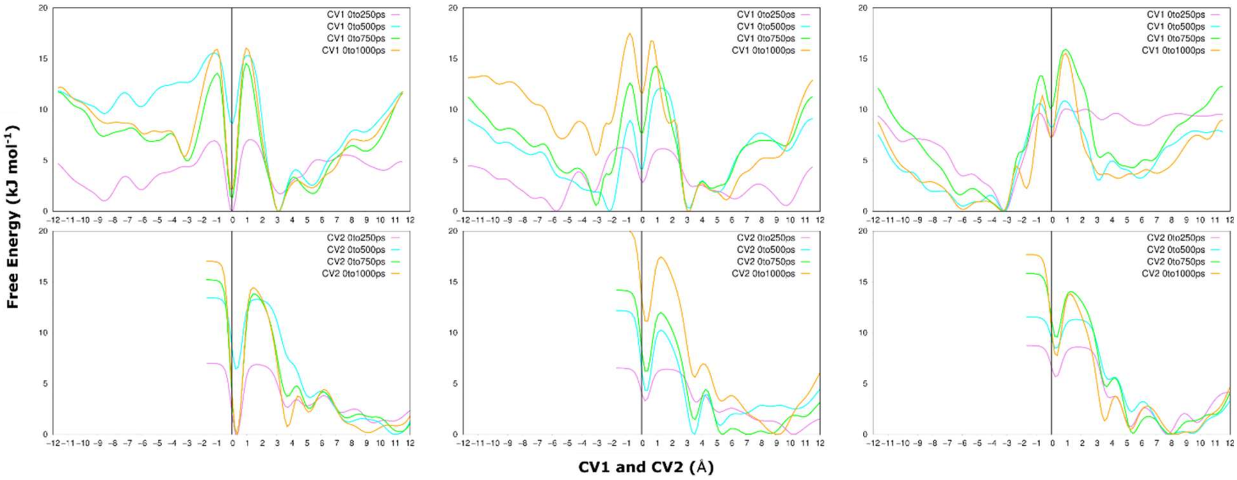

Figure S18. Free energy convergence (kJ mol^{-1}) of proton transport through benzenesulfonic (Ph-SO_3H) covalently functionalized graphene nanopore system along CV1 and CV2 (Å), top and bottom, respectively. To assess the convergence of the metadynamics simulations, each free energy profile is extracted after 0.25 ns (violet line), 0.5 ns (cyan

line), 0.75 ns (green line) and 1.0 ns (orange line) of each simulation, with a deposition stride every 25 fs, and the global minimum is set to zero in all profiles.

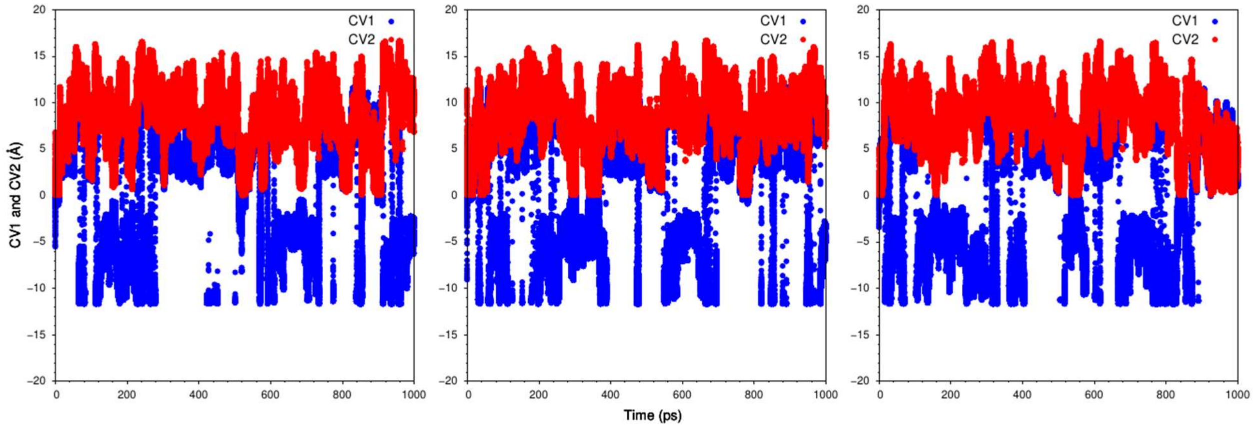

Figure S19. Time evolution of the Collective Variables CV1 (Å) and CV2 (Å) in blue and red lines, respectively, for each of the three final independent simulations for graphene nanopore functionalized with a benzenesulfonic (Ph-SO_3H) group.

S2.6 Proton and sodium cation selectivity for graphene nanopore covalently functionalized with Ph-SO_3H, or Ph-COOH, or Ph-OH, in aqueous environment.

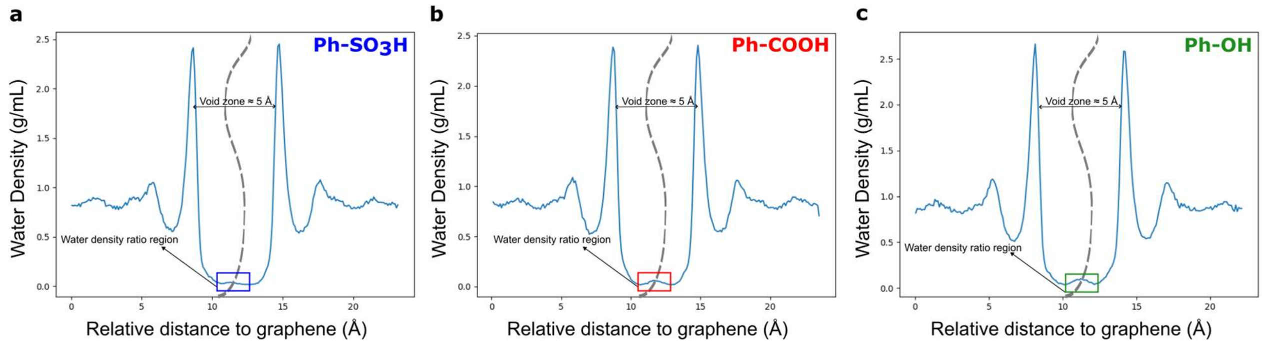

Figure S20. Density profiles (g/mL) of water along the relative distance to the graphene (Å) for the **a**, benzenesulfonic (Ph-SO_3H), **b**, benzoic (Ph-COOH), and **c**, phenol (Ph-OH) covalent functionalized graphene nanopore systems. The void zone of ~ 5 Å and the region for the water density ratio estimations have been highlighted. The graphene layer is represented with a gray dashed line by way of illustration.

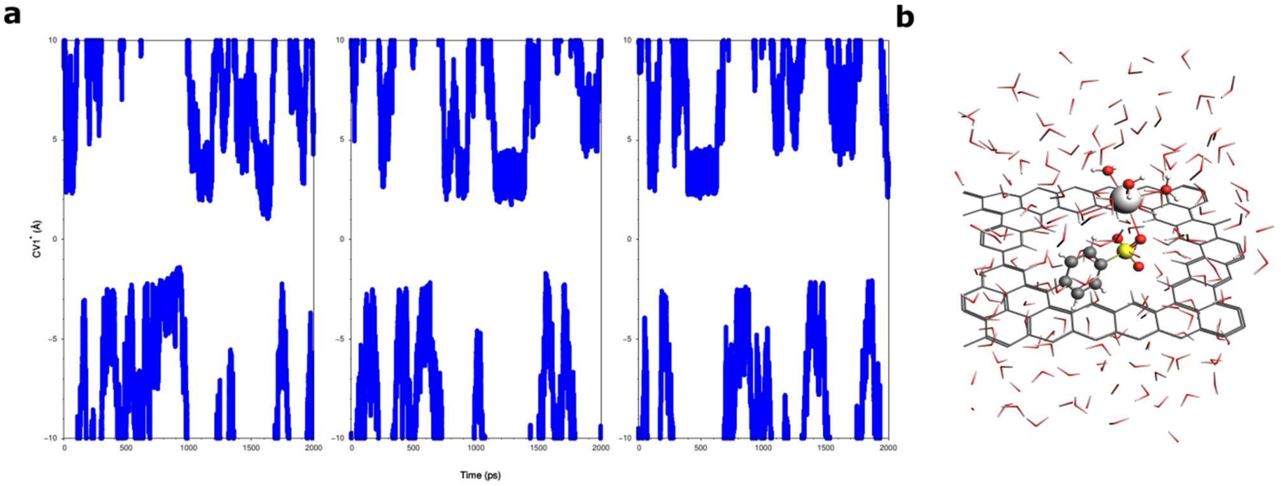

Figure S21. **a**, Time evolution of the Collective Variable CV1* (Å) for the three final independent simulations of the benzenesulfonic covalent functionalized (Ph-SO_3H) graphene nanopore system with a sodium cation in the water bulk. **b**, Representative configuration for the benzenesulfonic functionalized graphene nanopore system. The benzenesulfonic group, the sodium cation (Na^+) and the water molecules involved in its first solvation shell are represented by balls and sticks: carbon, oxygen, sulfur, hydrogen, and the sodium cation are colored in grey, red, yellow, white, and light grey, respectively.

REFERENCES

(1) Ganeshan, K.; Shin, Y. K.; Osti, N. C.; Sun, Y.; Prenger, K.; Naguib, M.; Tyagi, M.; Mamontov, E.; Jiang, D.; Van Duin, A. C. T. Structure and Dynamics of Aqueous Electrolytes Confined in 2D-TiO_2/$Ti_3C_2T_2$ MXene Heterostructures. *ACS Appl. Mater. Interfaces* **2020**, *12* (52), 58378–58389. https://doi.org/10.1021/acsami.0c17536.

(2) Fortunato, J.; Shin, Y. K.; Spencer, M. A.; Van Duin, A. C. T.; Augustyn, V. Choice of Electrolyte Impacts the Selectivity of Proton-Coupled Electrochemical Reactions on Hydrogen Titanate. *J. Phys. Chem. C* **2023**, *127* (25), 11810–11821. https://doi.org/10.1021/acs.jpcc.3c01057.

(3) Díaz Leines, G.; Ensing, B. Path Finding on High-Dimensional Free Energy Landscapes. *Phys. Rev. Lett.* **2012**, *109* (2), 020601. https://doi.org/10.1103/PhysRevLett.109.020601.

(4) Pérez De Alba Ortíz, A.; Tiwari, A.; Puthenkalathil, R. C.; Ensing, B. Advances in Enhanced Sampling along Adaptive Paths of Collective Variables. *The Journal of Chemical Physics* **2018**, *149* (7), 072320. https://doi.org/10.1063/1.5027392.

(5) Thompson, A. P.; Aktulga, H. M.; Berger, R.; Bolintineanu, D. S.; Brown, W. M.; Crozier, P. S.; In 'T Veld, P. J.; Kohlmeyer, A.; Moore, S. G.; Nguyen, T. D.; et al. LAMMPS - a Flexible Simulation Tool for Particle-Based Materials Modeling at the Atomic, Meso, and Continuum Scales. *Computer Physics Communications* **2022**, *271*, 108171. https://doi.org/10.1016/j.cpc.2021.108171.

(6) Bussi, G.; Donadio, D.; Parrinello, M. Canonical Sampling through Velocity Rescaling. *Journal of Chemical Physics* **2007**, *126* (1). https://doi.org/10.1063/1.2408420.

(7) *Ionization Constants of Heteroatom Organic Acids, Michigan State University, https://www2.chemistry.msu.edu/faculty/reusch/virttxtjml/acidity2.htm.* https://www2.chemistry.msu.edu/faculty/reusch/virttxtjml/acidity2.htm.

(8) Michaud-Agrawal, N.; Denning, E. J.; Woolf, T. B.; Beckstein, O. MDAnalysis: A Toolkit for the Analysis of Molecular Dynamics Simulations. *J. Comput. Chem.* **2011**, *32* (10), 2319–2327. https://doi.org/10.1002/jcc.21787.

(9) Gowers, R.; Linke, M.; Barnoud, J.; Reddy, T.; Melo, M.; Seyler, S.; Domański, J.; Dotson, D.; Buchoux, S.; Kenney, I.; et al. MDAnalysis: A Python Package for the Rapid Analysis of Molecular Dynamics Simulations; Austin, Texas, 2016; pp 98–105. https://doi.org/10.25080/Majora-629e541a-00e.

(10) Achtyl, J. L.; Unocic, R. R.; Xu, L.; Cai, Y.; Raju, M.; Zhang, W.; Sacci, R. L.; Vlassiouk, I. V.; Fulvio, P. F.; Ganesh, P.; et al. Aqueous Proton Transfer across Single-Layer Graphene. *Nat Commun* **2015**, *6* (1), 6539. https://doi.org/10.1038/ncomms7539.

(11) Shi, L.; Xu, A.; Chen, G.; Zhao, T. Theoretical Understanding of Mechanisms of Proton Exchange Membranes Made of 2D Crystals with Ultrahigh Selectivity. *J. Phys. Chem. Lett.* **2017**, *8* (18), 4354–4361. https://doi.org/10.1021/acs.jpclett.7b01999.

(12) Shi, L.; Xu, A.; Cheng, Y. Ether-Group-Mediated Aqueous Proton Selective Transfer across Graphene-Embedded 18-Crown-6 Ether Pores. *J. Phys. Chem. C* **2019**, *123* (45), 27429–27435. https://doi.org/10.1021/acs.jpcc.9b09715.

(13) Swart, M.; Bickelhaupt, F. M. Proton Affinities of Anionic Bases: Trends Across the Periodic Table, Structural Effects, and DFT Validation. *J. Chem. Theory Comput.* **2006**, *2* (2), 281–287. https://doi.org/10.1021/ct0502460.
